# Unlocking the Potential of Spheroids in Personalized Medicine: A Systematic Review of Seeding Methodologies

**DOI:** 10.3390/ijms26136478

**Published:** 2025-07-04

**Authors:** Karolina M. Lonkwic, Radosław Zajdel, Krzysztof Kaczka

**Affiliations:** 1 Clinic of General and Oncological Surgery, Medical University of Lodz, 92-213 Lodz, Poland; krzysztof.kaczka@umed.lodz.pl; 2 Mabion S.A., 95-050 Konstantynow Lodzki, Poland; 3 Department of Economic and Medical Informatics, University of Lodz, 90-214 Lodz, Poland; radoslaw.zajdel@uni.lodz.pl; 4 Department of Medical Informatics and Statistics, Medical University of Lodz, 90-645 Lodz, Poland

**Keywords:** spheroids, personalized medicine, drug screening, organoids, 3D models

## Abstract

Three-dimensional (3D) spheroid models have revolutionized in vitro cancer research by offering more physiologically relevant alternatives to traditional two-dimensional (2D) cultures. A systematic search identifies English-language studies on patient-derived cancer spheroids for drug screening, using defined inclusion and exclusion criteria, with data extracted on cancer type, culture methods, spheroid characteristics, and therapeutic responses. This manuscript evaluates the methods for spheroid formation and the cellular sources used, highlighting the diverse applications and preferences in this field. The five most investigated cancer origins for spheroid seeding are breast, colon, lung, ovary, and brain cancers, reflecting their clinical importance and research focus. Among seeding methodologies, forced-floating and scaffold-based methods predominate, demonstrating reliability and versatility in spheroid generation. Other techniques, including microfluidics, bioprinting, hanging drop, and suspension culture also play significant roles, each with distinct advantages and limitations. This review underscores the increasing use of spheroid models and the need for standardization in methodologies to enhance the reproducibility and translational potential in cancer research.

## 1. Introduction

In recent years, there has been a paradigm shift in biomedical research towards developing more clinically relevant models to study human physiology and diseases. Personalized medicine, which aims to tailor medical interventions based on individual patient characteristics, has gained prominence in the search for more effective and targeted treatments. Within this context, three-dimensional (3D) cell culture models, particularly spheroids, have emerged as valuable tools that bridge the gap between traditional two-dimensional (2D) cell cultures and in vivo studies. Spheroids, characterized by their spherically shaped cellular aggregates, exhibit enhanced physiological relevance by recreating the cell–cell and cell–matrix interactions found in native tissues. This article aims to explore the developing field of spheroid research, focusing on their potential applications in personalized medicine and elucidating the methodologies employed for spheroid seeding.

Traditional 2D monolayer culture cell culture systems are often inadequate in reproducing the complexity of in vivo tissue structures and functions. Spheroids, formed through the self-assembly of cells, provide a closer representation of the native tissue microenvironment. This three-dimensional architecture facilitates cell–cell interactions, nutrient gradients, and spatial organization, closely mimicking the in vivo conditions. As a result, spheroids have gained prominence as an advanced in vitro model that bridges the gap between conventional cell cultures and in vivo studies.

This systematic review aims to provide a comprehensive overview of the methodologies used for spheroid formation, with a particular focus on seeding techniques. Furthermore, it explores the types of cancers most represented by spheroid models and evaluates their potential utility in personalized medicine.

### 1.1. Models for In Vitro Studies

The concept of growing cells outside of the human body began in the early 20th century. In 1907, Ross Harrison obtained the first successful tissue culture by growing nerve fibers from frog embryos, which paved the way for cell culture techniques that have since become fundamental in biomedical research [1].

Traditional 2D monolayer cultures, such as those using HeLa cells established by George Otto Gey in 1951, have served as the fundamental for biomedical and cancer research [2]. The 2D monolayer culture technique dominated in vitro cancer research for much of the 20th century [3]. These models have enabled landmark discoveries in cancer biology, including the identification of oncogenic pathways and initial therapeutic screens. They have been the cornerstone of in vitro cancer research for decades due to their simplicity, cost-effectiveness, and adaptability to high-throughput screening. In these systems, cancer cells are grown on flat, rigid substrates, providing uniform exposure to nutrients, oxygen, and drugs. The standardized conditions in 2D cultures enable reproducible experiments and straightforward readouts, making them ideal for initial mechanistic studies and large-scale drug screening [4]. However, they fall short in replicating the complex microenvironments of in vivo tissues. Cells cultured in monolayer lack the spatial organization, oxygen and nutrient gradients, and dynamic cell–cell interactions characteristic of native tissues. Following isolation from the tissue and the subsequent transfer to 2D culture conditions, both cell morphology and division patterns are altered. Additionally, 2D culturing contributes to the loss of phenotypic diversity [5].

To overcome these limitations, the field has increasingly embraced 3D models, such as spheroids, organoids, and organ-on-chip platforms. Scientific reviews confirm that these models better simulate cell–cell and cell–matrix interactions, as well as the nutrient and oxygen gradients found in actual tumors. This advancement has significantly improved the study of tumor biology and drug resistance [6,7].

Each model possesses distinct advantages and limitations, and their suitability can vary depending on the experimental objectives. Given these differences, a critical evaluation of each model’s capabilities is essential. The choice between 2D and 3D systems should be guided by the biological question being addressed, the complexity of the experimental setup, and the translational relevance of the findings. Table 1 outlines the advantages, disadvantages, and future perspective for both 2D and 3D models. Moreover, Table 1 overviews the contexts in which each model may be most appropriately applied, emphasizing the importance of strategic model selection tailored to both research goals and practical considerations.

Two-dimensional and three-dimensional models are increasingly used in combination—either as an integrated approach or in parallel—each offering complementary advantages. While 2D cultures remain vital for high-throughput and preliminary screening due to their simplicity and reproducibility, 3D models better mimic tumor physiology, making them valuable for studying progression, metastasis, and therapy resistance. Recent advancements in in vitro modeling have extended beyond traditional 2D cultures toward more sophisticated 3D systems and hybrid approaches, aiming to better recapitulate the structural and biochemical complexity of native tissues [18]. For instance, hydrogel-based scaffolds with engineered microenvironments represent a promising direction in bridging the gap [19]. Moreover, hybrid systems bridge the gap between in vitro and in vivo conditions, combining the practicality of 2D with the biological relevance of 3D. This integrative approach strengthens cancer research, drug discovery, and personalized medicine [9,10,11].

However, there is an emerging consensus that research should transition from reliance on traditional 2D models to the adoption of more physiologically relevant 3D models [8,9].

### 1.2. Spheroids in Personalized Medicine

Within the broad category of 3D in vitro models, self-assembling cellular systems such as spheroids and organoids represent a distinct subgroup. These systems are generated through intrinsic cellular organization processes and represent a unique approach to mimicking physiological conditions. While both systems support cell-cell and cell-matrix interactions in a 3D environment, they differ significantly in terms of cellular complexity, structural organization, and functional potential.

Spheroids, first described in the 1970s, are multicellular aggregates that self-assemble into spherical structures [20]. They are typically composed of a single cell type or a limited number of cell types, often derived from cancer cell lines, stem cells, or primary tissues. Due to their relative simplicity, spheroids are primarily used for modeling tumor microenvironments, studying cancer biology, and evaluating drug penetration and cytotoxicity. They lack tissue-specific architecture and functional heterogeneity characteristic of native organs [21].

In contrast, organoids are cell-derived 3D structures that represent key structural and functional features of their tissue of origin. Generated from stem cells, organoids undergo self-organization and differentiation process that generate multiple, lineage-specific cell types arranged in a physiologically relevant architecture. As a result, organoids closely mimic the in vivo organization and function of organs such as the intestine, brain, kidney or liver [21].

Personalized medicine aims to customize medical interventions based on individual patient characteristics, and spheroids emerge as a powerful tool in this area. The inherent heterogeneity within patient populations can be more accurately represented using spheroids, enabling the development of personalized therapeutic strategies. By incorporating patient-specific cells into spheroid models, researchers can assess drug responses and tailor treatment regimens for improved clinical outcomes [22].

Spheroids have emerged as a pivotal three-dimensional (3D) in vitro model in cancer research, offering a significant leap forward in replicating the complexity of in vivo tumor biology. This enhanced physiological relevance makes spheroids an invaluable tool for advancing our understanding of cancer biology and improving preclinical evaluations of therapeutic agents [21,23].

The defining characteristic of spheroids is their ability to develop internal gradients of oxygen, carbon dioxide, nutrients, and metabolites. These gradients lead to the development of distinct cellular zones within the spheroid [21,23].

**Figure 1:**
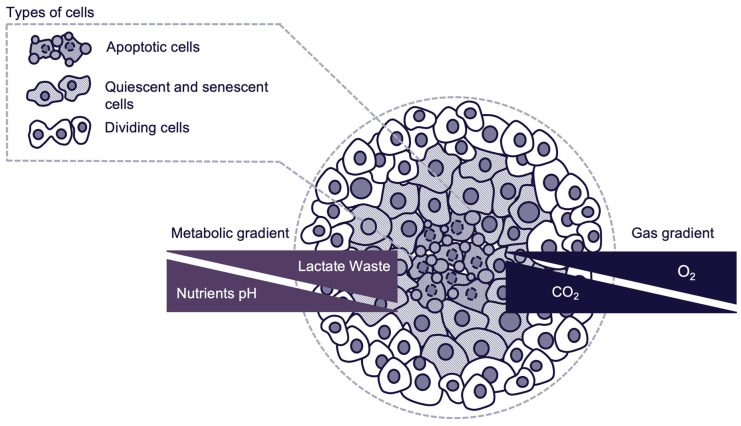
Schematic representation of the cellular structure of a spheroid.

Cellular zones of spheroids are visualized and presented in Figure 1. Three cellular zones of spheroids are as follows:

- Proliferative outer layer: Consisting of actively dividing cells, with high accessibility to oxygen and nutrients.
- Quiescent intermediate layer: Consisting of quiescent and senescent cells with reduced metabolic activity due to limited nutrient and oxygen availability.
- Hypoxic apoptotic core: Consisting of cells in an apoptotic state due to severe nutrient and oxygen deprivation. Core environment mimics what is observed in poorly vascularized tumor regions in vivo. Presence of hypoxic core depends on spheroid size, nutrient availability and culture environment.

This zonal architecture replicates the heterogeneous microenvironment of solid tumors, which is critical for studying tumor progression, metastasis, and resistance to therapies.

Spheroids are widely utilized in cancer research as robust models for examining critical biological processes in a three-dimensional context. They provide significant insights into tumor progression by enabling the detailed investigation of invasion, metastasis, and the interactions between the tumor and surrounding stroma. In the field of therapeutic screening, spheroids facilitate the evaluation of anticancer drug efficacy, penetration dynamics within the tumor microenvironment, and mechanisms of drug resistance. They are also essential for modeling tumor responses to hypoxia and radiation therapy. Additionally, spheroids play a significant role in immunotherapy research by supporting the study of immune cell–tumor interactions, thereby providing a more representative alternative to conventional two-dimensional culture models [22,23].

## 2. Materials and Methods

A comprehensive search was conducted in the PubMed database to identify relevant articles available until December 2024. The search utilized specific free words and Medical Subject Headings (MeSH) terms, including key terms such as ‘spheroid,’ ‘cancer,’ ‘drug,’ ‘patient-derived,’ and ‘tumor.’ The exclusion terms ‘co-culture’ and ‘xenograft’ were applied. Only articles published in English were considered for inclusion.

Inclusion criteria encompassed studies involving spheroids formed from patient-derived cancer cells, while exclusion criteria covered articles that did not meet these inclusion criteria such as reviews, case reports, and works focusing on the application or use of spheroids in areas other than drug screening.

Data extraction from the included articles comprised information on authors, publication year, title, cancer type, cell aggregation protocol.

The inclusion criterion of English-language publications may have introduced potential bias, excluding the relevant literature in other languages. Consequently, the findings should be interpreted cautiously, acknowledging the potential limitations associated with the language-based selection criteria.

## 3. Results and Discussion

### 3.1. Database Screening

**Figure 2:**
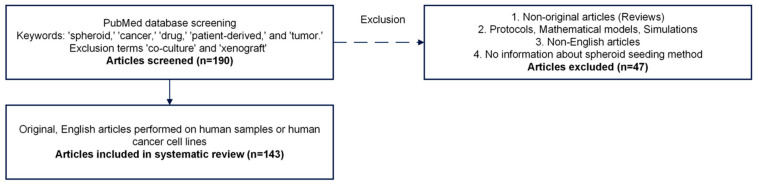
Flowchart of the study selection process for the systematic review, including inclusion and exclusion criteria, and the final number of studies selected.

Of the 190 articles retrieved from PubMed on spheroid seeding from human cancer cells for drug sensitivity screening, only 143 articles were evaluated for eligibility based on the search strategy outlined in the Materials and Methods section. The selection process is illustrated in Figure 2. The first step involved screening the PubMed database using specific keywords and exclusion terms, which yielded 190 articles. Of these, 47 were excluded based on predefined criteria. A total of 143 original, English-language studies conducted on human samples or human cancer cell lines were included.

### 3.2. Systematic Review

Table 2 displays the selected 143 articles along with their fundamental details, including the following: first author and publication year, cancer type by type of tissue in which cancer originates (histological type) and by primary site, and spheroid seeding method.

#### 3.2.1. Source of Spheroids

**Figure 3:**
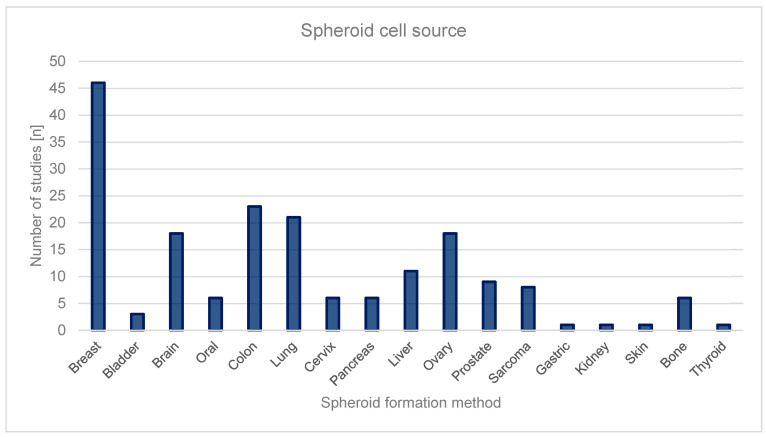
Distribution of cell sources used for spheroid generation presented as a column graph. The figure summarizes the number of studies reporting each cell source, as identified though the systematic review.

Spheroids derived from various cancer types are extensively utilized in research to mimic in vivo tumor characteristics, providing insights into diverse cancer-specific processes. The ability to generate spheroids from a variety of cell sources, including patient-derived tumor cells, further enhances their value in studying personalized therapeutic responses. In the present systematic review, the most common cell sources of spheroids were identified. Figure 3 illustrates the distribution of the included studies based on the tissue origin of the spheroid cell sources. The most frequently represented cancer type was breast cancer, accounting for 46 studies. This was followed by colon (23 studies), lung (20 studies), and brain (17 studies). Ovarian cancer spheroids were formed in 17 studies. Other cancer types, such as liver, cervix, pancreas, prostate, and sarcoma, were represented in fewer than 15 studies each. Less commonly studied sources included gastric, bladder, kidney, skin, bone, and thyroid tissues, each with fewer than six studies. This distribution highlights a predominance of spheroid models derived from breast and gastrointestinal cancers, suggesting a focus on these tumor types in current 3D culture-based research.

Table 3 summarizes that the five most investigated cancer origins associated with spheroids are breast (*n* = 46, accounting for 24.6% of all investigated cancers), followed by colon (*n* = 23, 12.3%), lung (*n* = 21, 10.7%), ovary (*n* = 19, 9.6%), and brain (*n* = 18, 9.6%).

##### Breast Cancer

Spheroids derived from breast cancer cells represent one of the most extensively studied in vitro models, reflecting the prominence of breast cancer as both a clinical challenge and a leading research focus. A systematic review of the literature revealed that breast cancer cell lines and patient-derived cells were the most frequently used sources for spheroid generation.

Breast cancer spheroid models facilitate the exploration of unique tumor characteristics (e.g., variations in growth dynamics, gene expression profiles, and interactions with the tumor microenvironment) and aspects of cancer biology (e.g., immortality, telomerase activation, antiapoptotic strategy) [30,38,58,167].

Moreover, this approach facilitates the exploration of therapies targeting estrogen-metabolizing enzymes and receptors, enabling the discovery of novel treatments that may prevent tumor initiation or inhibit cancer growth [36].

Additionally, this methodology supports the development of patient-specific drugs, thereby aligning with the principles of precision medicine to optimize therapeutic outcomes for individual breast cancer patients [69].

##### Colon Cancer

Colon cancer is the second most common source of cells used for spheroid generation, reflecting its critical role in cancer research.

To enhance the utility of human 3D colorectal cancer spheroid models in preclinical drug assessments, there is a need for standardized and validated methodologies. While monoculture spheroids are useful for high-throughput drug screening due to their simplicity, spheroids provide deeper insights into tumor biology and chemoresistance mechanisms, offering a more accurate preclinical tool for evaluating therapeutic efficacy and developing new drug candidates [42,168].

The 3D cultures still face challenges in clinical implementation, and advancements in co-culture techniques, addressing tumor heterogeneity, and improving laboratory protocols are essential for enhancing reproducibility and drug testing reliability in colorectal cancer research [169]. Moreover, collecting cells during biopsy from different tumor sites might provide a more comprehensive representation of tumor subclones, offering greater insight into the tumor’s diverse properties and improving the accuracy of preclinical models for drug testing and personalized medicine [169].

##### Lung Cancer

Lung cancer represents the third most common source of cells for generating spheroids. Lung cancer-derived spheroids are utilized to investigate key aspects of tumor biology.

Lung cancer spheroids are particularly valuable for studying the progression of both non-small-cell lung cancer (NSCLC) and small-cell lung cancer (SCLC), which differ significantly in their biological behavior and response to therapy [170,171].

Therapeutically, lung cancer spheroids serve as platforms for evaluating the efficacy of novel anticancer agents, including small molecules, biologics, and combination therapies. Their three-dimensional structure facilitates studies of drug delivery systems aimed at overcoming barriers such as limited penetration into solid tumors [50,57,59,77,87,129].

##### Ovarian Cancer

Ovarian cancer is the fourth most common source of cells used for spheroid generation. Spheroids derived from ovarian cancer cells are utilized in research to study the processes central to ovarian cancer pathology, such as peritoneal metastasis, chemoresistance, and interactions with the tumor microenvironment. Given the propensity of ovarian cancer to spread via the peritoneal cavity through multicellular aggregates, spheroids serve as a physiologically relevant model to replicate these metastatic behaviors in vitro [41,62,86,116,117].

Moreover, patient-derived ovarian cancer spheroids are increasingly used for precision oncology, allowing for the evaluation of personalized therapeutic strategies tailored to the molecular profiles of individual tumors [123,159].

##### Brain Cancer

Brain cancers, including glioblastoma and other gliomas, rank as the fifth most common source of cells used for spheroid generation.

These three-dimensional models are crucial for studying the unique microenvironment and invasive properties of brain tumors, which are characterized by their aggressive behavior and resistance to standard therapies. Brain cancer-derived spheroids closely mimic the in vivo conditions of brain tumors, providing insights into key processes such as tumor invasion, therapeutic resistance, and interactions with the extracellular matrix (ECM) [73,84,103]. Glioblastoma-derived spheroids are among the most studied in this category. They are particularly valuable for investigating the highly invasive nature of glioblastoma cells, which infiltrate the surrounding healthy brain tissue, making complete surgical resection nearly impossible [34,114].

Recent advancements include patient-derived brain cancer spheroids, which preserve the genetic and phenotypic heterogeneity of primary tumors. These models are increasingly used for personalized medicine, enabling the testing of individualized therapeutic regimens in a controlled in vitro setting [30,73,157].

#### 3.2.2. Spheroid Seeding Methods

The successful implementation of spheroids in personalized medicine relies on robust and reproducible methodologies for their generation. The generation of three-dimensional (3D) spheroids as in vitro models requires the careful consideration of seeding methods to ensure reproducibility, scalability, and physiological relevance. A variety of techniques have been developed to create spheroids, ranging from traditional methods to advanced approaches incorporating cutting-edge technologies. 

In conclusion, this article aimed to provide a thorough understanding of the methodologies employed in spheroid seeding and highlighted the manifold applications of spheroids in advancing personalized medicine.

**Figure 4:**
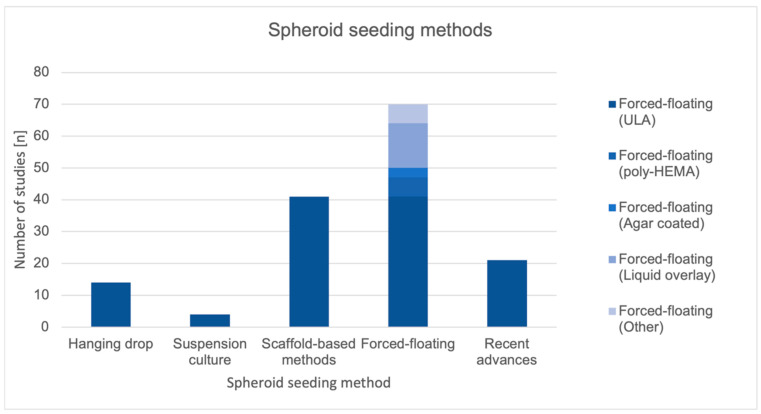
Distribution of spheroid seeding methods used for spheroid generation presented as a column graph. The figure summarizes the number of studies reporting each seeding method, as identified though the systematic review.

Figure 4 presents the distribution of the spheroid seeding methods utilized across the included studies. The most employed approach was the forced-floating method, with over 70 studies utilizing various subtypes of this technique. Among these, ultra-low attachment (ULA) plates were the most frequently used, followed by poly-HEMA-coated surfaces, agar-coated wells, liquid overlay, and other less common variations. Scaffold-based methods were also widely applied, appearing in 40 studies. In contrast, the hanging drop technique and suspension culture were used less frequently, with approximately 13 and 4 studies, respectively. A smaller but notable number of studies employed recently developed or advanced methods, indicating ongoing innovation in spheroid formation strategies. This distribution underscored the dominance of forced-floating techniques in current spheroid culture protocols, while also reflecting methodological diversity and emerging alternatives.

Table 4 summarizes the five most investigated spheroid seeding methodologies; the most common were forced-floating (*n* = 70, accounting for 31.8% of studies), scaffold-based methods (*n* = 41, 18.6%), recent advances (*n* = 21, 9.5%), hanging drop (*n* = 14, 6.4%) and suspension culture (*n* = 4, 1.8%). A summary of the systematic review results is presented.

**Figure 5:**
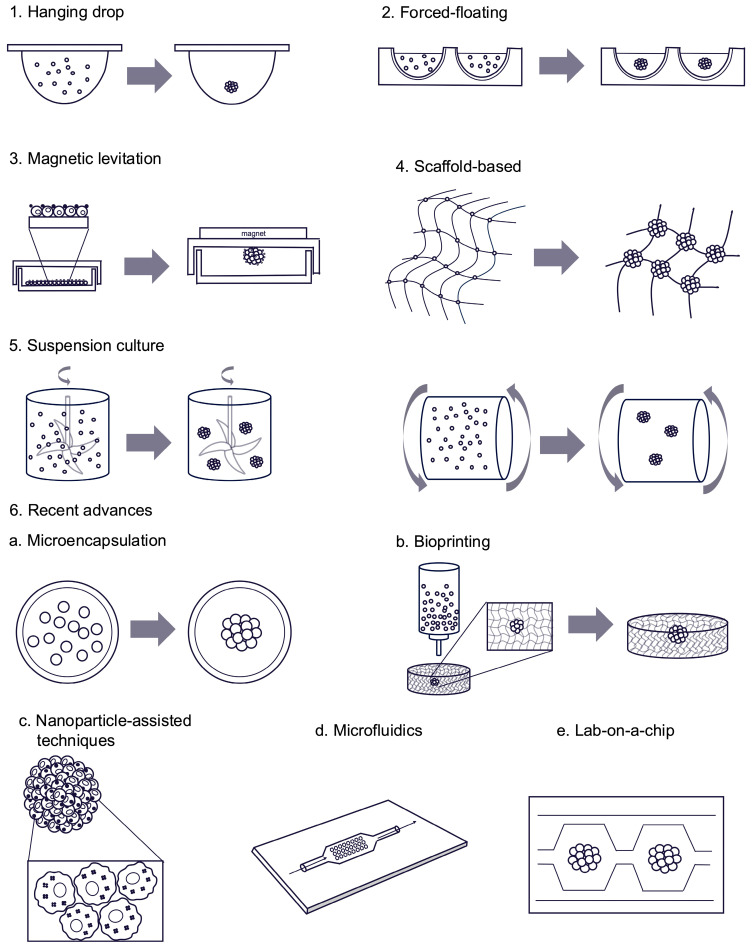
Overview of techniques used for spheroid formation. The figure provides a graphical representation of the various seeding methods employed in the generation of spheroids, as identified in the systematic review.

Based on the systematic review, a summary of the most common spheroid seeding methods is illustrated in Figure 5. The figure visualizes the most utilized spheroid seeding methods such as hanging drop (1), forced-floating (2), magnetic levitation (3), scaffold-based (4), suspension culture (5), and recent scientific advances (6) including: microencapsulation (6a), bioprinting (6b), nanoparticle-assisted techniques (6c), microfluidics (6d) and lab-on-a-chip (6e).

##### Forced-Floating

The forced-floating method is the most employed technique for spheroid formation due to its simplicity and scalability. This approach uses non-adhesive surfaces to prevent cell attachment, encouraging cells to aggregate and form three-dimensional (3D) spheroids. The technique involves seeding cells in multi-well plates that have been treated to inhibit surface adhesion, either through coating with low-attachment materials like poly-HEMA (Poly(2-hydroxyethyl methacrylate)) or using ultra-low attachment (ULA) culture plates designed specifically for this purpose. In the absence of adhesion sites, cells naturally aggregate in the medium, forming spheroids under the influence of gravity and intercellular interactions. This process begins with the preparation of a single-cell suspension of the desired cell density, typically ranging from 10^3^ to 10^5^ cells per well, depending on the type of cells and the intended spheroid size. The cell suspension is then distributed into the wells of the plate. After 24–96 h, depending on the cell type and experimental conditions, the cells self-assemble into compact spheroids [31,109,148,163].

The forced-floating method is particularly advantageous for producing uniform spheroids with consistent size and morphology, making it suitable for high-throughput applications such as drug screening and toxicity assays [11,39,63,65,100,148]. Additionally, this method does not require specialized equipment beyond the plates or coatings, making it accessible for most laboratories. One of the significant benefits of the forced-floating method is its compatibility with automated systems, allowing for large-scale spheroid generation and analysis. [11,63,148]. However, the method has certain limitations. The reliance on non-adhesive surfaces can lead to variability in spheroid integrity and size if the cell density or culture conditions are not carefully optimized [11,63]. Additionally, long-term culture may be constrained by limited nutrient and oxygen diffusion, necessitating periodic medium exchange or supplementation with perfusion systems [65].

##### Scaffold-Based

The scaffold-based method for spheroid seeding is the second most utilized method for generating three-dimensional (3D) cellular aggregates. This method uses biomaterials, known as scaffolds, that mimic the extracellular matrix (ECM) to provide the structural support and environment conducive to cell adhesion, proliferation, and aggregation into spheroids. Scaffolds can be fabricated from a wide range of materials, including natural polymers such as collagen, gelatin, and alginate, as well as synthetic polymers like PLGA (poly(lactic-co-glycolic acid)) and PEG (polyethylene glycol). The process typically begins with the preparation of the scaffold material, which may be in the form of hydrogels, porous matrices, or microcarriers. Cells are then seeded onto or encapsulated within the scaffold. Once seeded, the cells interact with the scaffold material and with one another, eventually forming spheroid structures. The scaffold not only facilitates cell aggregation, but it also supports nutrient and oxygen diffusion, which is crucial for maintaining cell viability and function in 3D cultures [15,148,172].

Scaffold-based methods offer significant advantages, including the ability to recreate a more physiologically relevant microenvironment compared to non-adhesive-based techniques. The structural and biochemical properties of the scaffold can be engineered to closely mimic the in vivo ECM, supporting the growth and differentiation of specific cell types [15]. This makes the method particularly suitable for modeling complex tissues and for co-culture systems involving multiple cell types [173]. Additionally, scaffold-based systems are compatible with long-term culture, as the scaffold provides a sustained environment for nutrient and waste exchange [15]. However, there are limitations to this approach. The use of scaffolds introduces variability in spheroid size and shape, depending on the uniformity of the material and the seeding protocol [15]. The composition and mechanical properties of the scaffold can also influence cellular behavior, which may complicate the interpretation of results [15]. Furthermore, the cost and complexity of scaffold fabrication, particularly for advanced synthetic materials, may be a barrier for some applications [11,15].

##### Hanging Drop

The hanging drop method is robust and is the third most utilized technique for generating spheroids in vitro, particularly valued for its ability to produce uniform and physiologically relevant cellular aggregates. This method capitalizes on gravity-driven cellular self-assembly within droplets of culture medium, facilitating interactions that mimic those found in vivo. To implement this approach, a cell suspension of the desired density is prepared, often ranging from 10^2^ to 10^4^ cells per droplet. Droplets, typically 20–50 μL in volume, are then dispensed onto the inner surface of an inverted Petri dish lid. The droplets are retained by surface tension, allowing them to remain suspended. This setup is placed over a dish containing a hydrating agent, such as phosphate-buffered saline (PBS) or water, to maintain humidity and prevent evaporation during incubation. After 24 to 72 h under standard culture conditions, the suspended cells settle at the bottom of the droplets and aggregate into spheroids through intercellular adhesion and natural cell–cell interactions [24,41,60,120,122,151].

The hanging drop method is particularly advantageous due to its simplicity, low cost, and minimal equipment requirements [11,60,174]. This method offers significant benefits, including enhanced cellular aggregation and adhesion driven by gravitational forces, which minimize mechanical damage to spheroids. It also allows for precise control over the spheroid size and cell composition by adjusting the initial cell density and droplet volume [11,60]. However, this technique has limitations, such as the potential disruption of spheroids during transfer to conventional culture plates due to mechanical stress. Moreover, it is labor-intensive and not easily scalable for high-throughput applications, as each droplet must be individually prepared and managed. Additionally, nutrient and waste exchange are limited by the small volume of medium, necessitating careful monitoring to maintain spheroid viability [11,120,140,175].

##### Suspension Culture

The suspension culture method is the fourth most utilized approach for generating spheroids in in vitro studies, particularly in cancer research, developmental biology, and drug discovery. This technique relies on culturing cells in a liquid medium without a solid substrate, allowing them to aggregate and form spheroids due to intercellular adhesion and natural aggregation tendencies. Typically, the suspension culture is conducted in culture vessels, such bioreactors or spinner flasks, to prevent cell adhesion to the container surface and promote spheroid formation [148,175]. The process begins with the preparation of a single-cell suspension at a defined density, which is a critical parameter for achieving a uniform spheroid size and morphology. These vessels prevent cells from adhering to the surface and maintain them in suspension. Spinner flasks or bioreactors are dynamic systems, where gentle agitation or rotation keeps the cells suspended and evenly distributed, which can enhance the uniformity of spheroid formation and improve the mass transport of nutrients and oxygen [30,148,175].

The suspension culture method offers several advantages. It is relatively simple and cost-effective, requiring minimal specialized equipment beyond vessels. The method is highly versatile, accommodating various cell types and allowing for the easy incorporation of co-culture systems to model complex cell–cell interactions, such as those between tumor and stromal cells. Furthermore, the suspension culture can be adapted for high-throughput applications, making it suitable for large-scale drug screening and toxicity testing [11,175,176]. Despite its strengths, the suspension culture method has limitations. Nutrient and oxygen diffusion can be inadequate in larger spheroids, leading to hypoxic or necrotic cores. This limitation necessitates the careful control of spheroid size and medium composition to maintain viability. Additionally, while the method is relatively straightforward, achieving consistent spheroid size and morphology can be challenging without precise control over cell seeding density and culture conditions [11,175,176]. Moreover, prolonged culture durations may require frequent medium changes or the use of perfusion systems to sustain spheroid health [11,175,176].

##### Magnetic Levitation

The magnetic levitation method is a less frequently used approach to spheroid formation. This method utilizes leveraging magnetic fields to promote cellular aggregation and 3D structure development. This technique involves the use of magnetic nanoparticles that are internalized by cells through incubation. The nanoparticles are typically composed of biocompatible materials such as iron oxide and may be functionalized with ECM proteins or other molecules to enhance cellular uptake and minimize toxicity. After the cells have internalized the nanoparticles, they are exposed to a magnetic field, which forces them to aggregate and suspend in the culture medium. The magnetic field enables the controlled formation of compact spheroids by facilitating cell–cell and cell–ECM interactions [175,177]. Magnetic levitation offers several advantages. It allows for rapid and reproducible spheroid generation and provides a high degree of control over spheroid size and structure. Additionally, this method supports the formation of co-culture spheroids by enabling the simultaneous aggregation of different cell types, which is particularly useful for modeling tumor–stroma or tumor–immune cell interactions. The technique also facilitates the incorporation of ECM components, improving the physiological relevance of the spheroid microenvironment. Furthermore, magnetic levitation is amenable to high-throughput applications and can be easily scaled up for drug screening or other large-scale studies. Despite its advantages, the method has limitations. The requirement for magnetic nanoparticles introduces potential concerns regarding biocompatibility and cellular toxicity, particularly for long-term studies. Additionally, nanoparticle uptake among cells can vary, potentially leading to inconsistencies in spheroid formation. The cost of magnetic nanoparticles and specialized magnetic devices may also be a barrier for some laboratories [11,175,177].

##### Recent Advances in Spheroid Seeding Methods

Recent advances in spheroid seeding include novel techniques that have emerged in the past few years and do not fall within the conventional classifications of standard seeding methods. These approaches represent hybrid or innovative strategies that extend beyond traditional categories such as hanging drop or low-attachment culture. Recent advances in spheroid seeding methods are represented by microencapsulation, bioprinting, nanoparticle-assisted techniques, microfluidics, and lab-on-a-chip methods.

Microencapsulation involves embedding cells or spheroids within biocompatible hydrogels to recreate ECM-like environments. This technique enhances cell–cell interactions, shields cells from shear stress, and supports co-culture configurations. However, traditional methods often suffer from inconsistent spheroid loading, size variability, nutrient diffusion limitations, and difficulties in spheroid retrieval [165,178]. For example, Chan et al. developed a microfluidic double-emulsion system that directly generates uniform microencapsulated hepatocyte spheroids (<200 μm) within 4 h. Using an alginate–collagen hydrogel matrix, the encapsulated spheroids exhibited superior hepatic functions—albumin and urea secretion, and cytochrome P450 activity—over 24 days compared to alginate-only or collagen-sandwich cultures [179].

Bioprinting is a method utilizing 3D printing technologies, allowing for the precise placement of cells and biomaterials to replicate native tissue architecture. It offers exceptional control over spheroid organization and is particularly effective for high-throughput applications and heterotypic co-cultures. Limitations include the need for bioink optimization and the high costs of equipment. Bioprinting enables the spatially controlled deposition of cell-laden bioinks to organize spheroids into functional 3D tissues [180,181]. Extrusion-based bioprinting is an effective method for spheroid seeding, that fabricates tissue constructs by continuously dispensing bioink through a nozzle, allowing for the precise deposition of cell-laden materials. It is valued for its simplicity, scalability, and affordability, but shear stress during extrusion is a critical factor influencing cell viability and print quality [182]. Inkjet is a non-contact, droplet-based technique that deposits picolitre-sized droplets of bioink—comprising cells and biomaterials—onto substrates using thermal or piezoelectric actuation. This method offers high-resolution patterning and cost-effectiveness but is constrained by the requirement for low-viscosity bioinks, limiting the achievable cell densities and construct complexity [183,184].

Nanoparticle-assisted methods refer to a broader set—than magnetic levitation—of strategies in which nanoparticles are used to support or enhance various aspects of spheroid formation, without necessarily involving magnetic fields or levitation. This method precisely guides cell aggregation into spheroids by leveraging functionalized nanoparticles. Nanoparticles—ranging from magnetic to non-magnetic (e.g., gold, silica, polymer-based)—serve diverse roles such as carriers for bioactive agents, enhancers of cell adhesion, or modulators of the microenvironment. Unlike magnetic levitation, these techniques do not directly position or aggregate cells but rather provide biochemical or structural cues that facilitate spheroid development, improve viability, or guide differentiation. This method is highly versatile and can be tailored to specific experimental goals, including drug testing or tissue engineering. It offers reproducibility and integration with imaging or therapeutic applications but raises concerns about nanoparticle cytotoxicity and scalability [46,73,138,179].

Microfluidic and lab-on-a-chip are technologies of spheroid seeding that offer precise control over the microenvironment for spheroid formation, enabling uniformity in size and structure, and facilitating high-throughput applications [35,41,57,90,94,108,185]. An example of a microfluidic platform might be a microwell-based system. These systems utilize arrays of microwells to confine cells, promoting self-aggregation into spheroids. For instance, pyramidal microwells with a 90° tip angle have been shown to enhance the spheroid formation and cardiac differentiation of mouse embryonic stem cells [186].

##### Summary of Spheroid Seeding Method

A summary of the advantages and limitations of selected spheroid seeding methods is presented in Table 5.

## 4. Conclusions

This systematic review underscores the expanding role of three-dimensional (3D) cancer cell models, particularly spheroids, in advancing cancer research, drug development, and personalized medicine. Among the studies analyzed, breast cancer was the most frequently investigated source for spheroid formation, followed by colon, lung, ovarian, and brain cancers, reflecting their clinical relevance and research priority. Forced-floating and scaffold-based techniques emerged as the most employed methods due to their relative simplicity, reproducibility, and broad applicability across various cancer types. The hanging drop technique, although more labor-intensive, is gaining traction for its ability to produce physiologically relevant 3D structures.

Despite the progress made, establishing reproducible protocols tailored to different spheroid seeding techniques remains a significant challenge, limiting efforts toward standardization and the broader adoption of these models in therapeutic screening. Additionally, conventional spheroid models often fall short in replicating the structural and functional complexity of in vivo tumors, particularly with respect to tumor heterogeneity and microenvironmental interactions. The increasing application of patient-derived spheroids represents a promising development, offering models that better capture the genetic and phenotypic variability of individual tumors and supporting efforts toward more personalized therapeutic strategies.

### Future Perspectives

Future research should prioritize the development of standardized and reproducible protocols for spheroid generation, especially those compatible with high-throughput drug screening platforms. Integrating tumor heterogeneity—with patient-derived models and sampling from multiple tumor regions—will be essential to improve model relevance and predictive accuracy. Additionally, incorporating co-culture systems that include stromal, immune, and vascular components may better replicate the tumor microenvironment and enhance the physiological fidelity of in vitro models. Advancements in the automation, miniaturization, and real-time monitoring of spheroid cultures will further support the translation of 3D cancer models into clinically meaningful applications, ultimately contributing to the development of more effective, patient-tailored cancer therapies.

## Figures and Tables

**Table 1:** Advantages, limitations, and potentials of 2D and 3D models.

	2D Models	3D Models
Advantages	Simplicity and ease of use [5,8]. Cost-effectiveness and availability [5,9,10]. High-throughput capability [7,8].	Closer mimicry of in vivo conditions [10]. Replication of tumor microenvironment and cellular interactions (e.g., cell–cell and cell–matrix interactions, hypoxic core) [7,8,10,11,12,13]. Better simulation of drug penetration and resistance (drug diffusion barriers and heterogeneous cellular responses) [7,8,10,11,12]. Representation of gene and protein expression profile that reflects tumors [10,12,14].
Limitations	Lack of physiological relevance [8,11,13]. Limited replication of cell–cell and cell–matrix interactions [10,11,13]. Fails to simulate gradients of oxygen, nutrients, and metabolites [5].	Higher cost and technical complexity [5,9,10,12]. Longer culture time [5]. Scalability challenges [15]. Reproducibility issues in some systems [5,9].
Potentials	Effective for initial drug screening and basic mechanistic studies [12,15]. Suitable for large-scale studies with consistent and reproducible outputs [12].	Ideal for studying tumor progression, metastasis, and resistance mechanisms [11,16]. Promising for personalized medicine applications, including patient-derived models [17].

**Table 2:** Articles selected for systematic review.

Reference	Cancer Primary Site (Histological Type)	Cell Line	Spheroid Formation Method
Agastin S, 2011 [24]	Colon (adenocarcinoma), Breast (adenocarcinoma)	Colo205, MDA-MB-231	Hanging drop
Alhasan L, 2016 [25]	Breast (carcinoma)	BT-474	Scaffold-based methods
An HJ, 2020 [26]	Kidney (carcinoma)	A498	Scaffold-based methods
Árnadóttir SS, 2018 [27]	Colon	Patient-derived	Forced-floating (not defined)
Baek N, 2016 [28]	Prostate (carcinoma), Bone (neuroblastoma), Lung (carcinoma), Cervix (adenocarcinoma), Bone (osteosarcoma)	DU145, SH-SY5Y, A549, HeLa, HEp2, 0U-2OS	Forced-floating (agar-coated plates)
Barone RM, 1981 [29]	Colon (adenocarcinoma)	HT-29	Suspension culture
Bartholomä P, 2005 [30]	Breast (carcinoma)	T-47D	Suspension culture
Boo L, 2020 [31]	Breast (adenocarcinoma)	MCF-7	Forced-floating (agar-coated plates)
Brooks EA, 2019 [32]	Ovary (adenocarcinoma)	Patient-derived, AU565, BT549, SKOV-3	Scaffold-based methods
Bruns J, 2022 [33]	Brain (glioblastoma)	Patient-derived, U87	Scaffold-based methods
Calori IR, 2022 [34]	Brain (glioblastoma, medulloblastoma)	U87, T98G, A172, UW473	Forced-floating (ultra-low attachment (ULA))
Chang S, 2022 [35]	Breast (adenocarcinoma)	MCF-7	Recent advances
Chen G, 2022 [36]	Breast (adenocarcinoma)	MCF-7	Forced-floating (ULA)
Chen MC, 2010 [37]	Breast (melanoma)	LCC6/Her-2	Recent advances
Chen Z, 2021 [38]	Breast (adenocarcinoma)	MDA-MB-231	Forced-floating (ULA)
Cheng V, 2015 [39]	Brain (glioblastoma)	U87, U251	Forced-floating (ULA)
Close DA, 2022 [40]	Oral (squamous cell carcinoma)	Cal33, FaDu, UM-22B, OSC-19	Forced-floating (ULA)
Das T, 2013 [41]	Ovary (adenocarcinoma)	TOV112D	Hanging drop
Das V, 2016 [42]	Colon (carcinoma), Colon (adenocarcinoma), Bone (osteosarcoma), Cervix (adenocarcinoma), Colon (adenocarcinoma), Liver (carcinoma)	HCT116, HT29, U-2OS, HeLa, Caco-2, HepG2	Forced-floating (liquid overlay)
Das V, 2017 [43]	Colon (carcinoma)	HCT116	Forced-floating (liquid overlay)
De Angelis ML, 2018 [44]	Colon	Patient-derived	Forced-floating (ULA)
Dhamecha D, 2021 [45]	Lung (carcinoma), Bone (osteosarcoma)	A549, MG-63	Scaffold-based methods
Dias DR, 2016 [46]	Cervix (adenocarcinoma)	HeLa	Recent advances
Domenici G, 2021 [47]	Bone (sarcoma)	Patient-derived	Forced-floating (ULA)
Dufau I, 2012 [48]	Pancreas (adenocarcinoma)	Capan-2	Forced-floating (Poly(2-hydroxyethyl methacrylate) (poly-HEMA))
Eetezadi S, 2018 [49]	Ovary (carcinoma), Ovary (adenocarcinoma)	UWB1.289, UWB1.289+BRCA1, OV-90, SKOV3, PEO1, PEO4, COV362	Forced-floating (ULA)
Eguchi H, 2022 [50]	Lung (carcinoma)	A549	Forced-floating (ULA)
Eimer S, 2012 [51]	Brain (glioblastoma)	Patient-derived	Forced-floating (ULA)
El-Sadek IA, 2021 [52]	Breast (adenocarcinoma)	MCF-7	Forced-floating (ULA)
Enmon RM Jr, 2001 [53]	Prostate (carcinoma)	DU 145	Forced-floating (agar plates)
Flørenes VA, 2019 [54]	Skin (melanoma)	Patient-derived	Forced-floating (ULA)
Fu J, 2020 [55]	Liver (carcinoma), Prostate (carcinoma), Lung (carcinoma), Breast (adenocarcinoma)	HepG2, DU 145, A549, MCF-7, MDA-MB-231	Scaffold-based methods
Fu JJ, 2018 [56]	Prostate (carcinoma)	DU 145, LNCap	Scaffold-based methods
Gao Y, 2022 [57]	Lung (carcinoma)	A549	Recent advances
Gencoglu MF, 2018 [58]	Breast (adenocarcinoma), Breast (carcinoma), Prostate (carcinoma), Prostate (adenocarcinoma), Ovary (adenocarcinoma)	AU565, BT549, BT474, HCC 1419, HCC 1428, HCC 1806, HCC 1954, HCC 202, HCC 38, ZR75 1, HCC 70, LNCaPcol, PC3, SKOV3	Scaffold-based methods, Microwells, Suspension culture
Gendre DAJ, 2021 [59]	Lung (mesothelioma), Lung (adenocarcinoma)	H2052, H2052/484, H2452, LuCa1, LuCa61, LuCa62	Scaffold-based methods
Gheytanchi E, 2021 [60]	Colon (adenocarcinoma)	HT-29, Caco-2	Hanging drop, Forced-floating (poly-HEMA)
Goisnard A, 2021 [61]	Breast (carcinoma), Breast (adenocarcinoma)	SUM1315, MDA-MB-231, HCC1937, SW527, DU4475	Forced-floating (ULA)
Guo X, 2019 [62]	Ovary (adenocarcinoma), Colon (adenocarcinoma), Pancreas (carcinoma), Prostate (adenocarcinoma)	OVCAR3, SW620, PANC-1, PC3	Scaffold-based methods
Hagemann J, 2017 [63]	Oral (carcinoma)	FaDu, Cal27, UPCI-SCC-154	Forced-floating (ULA), Hanging drop
Han S, 2022 [64]	Liver	Patient-derived	Forced-floating (ULA)
Harmer J, 2019 [65]	Brain (glioblastoma)	U251, KNS42	Scaffold-based methods
Herter S, 2017 [66]	Colon (adenocarcinoma)	LS174T, LoVo	Hanging drop
Ho WY, 2012 [67]	Breast (adenocarcinoma)	MCF-7	Forced-floating (liquid overlay)
Ho WY, 2021 [68]	Breast (adenocarcinoma)	MCF-7	Scaffold-based methods
Hofmann S, 2022 [69]	Breast	Patient-derived	Forced-floating (ULA)
Hornung A, 2016 [70]	Colon (adenocarcinoma)	HT-29	Scaffold-based methods
Huang Z, 2020 [71]	Breast (adenocarcinoma)	MDA-MB-231	Scaffold-based methods
Jove M, 2019 [72]	Breast (adenocarcinoma), Colorectal (adenocarcinoma)	MCF-7, DLD-1	Scaffold-based methods
Ju FN, 2023 [73]	Brain (glioblastoma)	U87	Recent advances
Karamikamkar S, 2018 [74]	Breast (adenocarcinoma)	MCF-7	Scaffold-based methods
Karlsson H, 2012 [75]	Colon (carcinoma)	HCT-116	Forced-floating (ULA)
Karshieva SS, 2022 [76]	Colon (carcinoma), Liver (carcinoma)	HCT-116, Huh7	Forced-floating (ULA)
Kato EE, 2021 [77]	Lung (carcinoma)	A549	Hanging drop
Kim CH, 2020 [78]	Liver (carcinoma)	HepG2	Recent advances
Ko J, 2019 [79]	Brain (glioblastoma)	U87	Scaffold-based methods
Kochanek SJ, 2019 [80]	Oral (carcinoma)	Cal33, Cal27, FaDu, UM-22B, BICR56, OSC-19, PCI-13, PCI-52, Detroit-562, UM-SCC-1, and SCC-9	Forced-floating (ULA)
Kochanek SJ, 2020 [81]	Oral (carcinoma)	Cal33, FaDu, UM-22B, BICR56, OSC-19	Forced-floating (ULA)
Koshkin V, 2016 [82]	Breast (adenocarcinoma)	MCF-7	Scaffold-based methods
Kroupová J, 2022 [83]	Colon (adenocarcinoma)	HT-29	Forced-floating (not defined)
Kudláčová J, 2020 [84]	Brain (glioblastoma)	U87	Forced-floating (ULA)
Kumari P, 2017 [85]	Cervix (adenocarcinoma), Lung (carcinoma)	HeLa, A549	Scaffold-based methods
Lal-Nag M, 2017 [86]	Ovary (adenocarcinoma)	Hey-A8–GFP	Forced-floating (ULA)
Lama R, 2013 [87]	Lung (carcinoma)	H292	Scaffold-based methods
Landgraf L, 2022 [88]	Prostate (adenocarcinoma), Brain (glioblastoma)	PC-3, U87	Forced-floating (liquid overlay)
Le VM, 2016 [89]	Lung (carcinoma), Colon (carcinoma), Brain (glioblastoma)	95-D, HCT-116, U87	Scaffold-based methods
Lee SW, 2019 [90]	Lung (carcinoma)	A549	Recent advances
Lee Y, 2022 [91]	Lung (carcinoma)	H460, A549	Forced-floating (ULA)
Lemmo S, 2014 [92]	Breast (adenocarcinoma)	MDA-MB-231	Scaffold-based methods
Li M, 2019 [93]	Cervix (carcinoma)	C-33-A, DoTC2 4510	Forced-floating (ULA)
Lim W, 2018 [94]	Colon (carcinoma), Brain (glioblastoma)	HCT-116, U87	Recent advances
Lin ZT, 2021 [95]	Breast (adenocarcinoma)	MDA-MB-436	Scaffold-based methods
Liu X, 2021 [96]	Sarcoma	HS-SY-II	Recent advances
Lorenzo C, 2011 [97]	Pancreas (adenocarcinoma)	Capan-2	Forced-floating (poly-HEMA)
Luan Q, 2022 [98]	Lung (adenocarcinoma), Lung (carcinoma)	HCC4006, H1975, A549	Scaffold-based methods, Forced-floating (ULA)
Madsen NH, 2021 [99]	Breast (adenocarcinoma), Colon (adenocarcinoma), Pancreas (carcinoma)	MCF-7, HT-29, PANC-1, MIA PaCa-2	Forced-floating (ULA)
Marshall SK, 2022 [100]	Bone (osteosarcoma)	MG-63	Forced-floating (ULA)
Maruhashi R, 2018 [101]	Lung (carcinoma)	A549	Forced-floating (ULA)
Melnik D, 2020 [102]	Thyroid (carcinoma)	FTC-133	Suspension culture
Molyneaux K, 2021 [103]	Brain (glioblastoma)	LN229, U87, Gli36	Forced-floating (not defined)
Monazzam A, 2007 [104]	Breast (adenocarcinoma)	MCF-7	Forced-floating (agar plates)
Morimoto T, 2023 [105]	Gastric	Patient-derived	Scaffold-based methods
Mosaad EO, 2018 [106]	Prostate (cancer), Prostate (carcinoma)	C42B, LNCaP	Recent advances
Mueggler A, 2023 [107]	Lung	Patient-derived	Scaffold-based methods
Nashimoto Y, 2020 [108]	Breast (adenocarcinoma)	MCF-7	Recent advances
Nigjeh SE, 2018 [109]	Breast (adenocarcinoma)	MDA-MB-231	Forced-floating (agar plates), Forced-floating (ULA)
Nittayaboon K, 2022 [110]	Colon (carcinoma)	PMF-k014	Forced-floating (poly-HEMA)
Ohya S, 2021 [111]	Prostate (carcinoma)	LNCaP	Forced-floating (ULA)
Oliveira MS, 2016 [112]	Breast (adenocarcinoma), Ovary (adenocarcinoma)	MCF-7/Adr, NCI/Adr	Forced-floating (liquid overlay)
Ono K, 2022 [113]	Oral (carcinoma)	SAS, HSC-3, HSC-4, OSC-19	Forced-floating (ULA)
Pampaloni F, 2017 [114]	Brain (glioblastoma)	U343	Forced-floating (liquid overlay)
Park MC, 2016 [115]	Brain (glioblastoma)	Patient-derived and PC14PE6, PC14PE6_LvBr3, D54, LN428, LN751, U251E4, U87E4, SN-12C, SNU-119, SNU-216, SNU-668, SNU-719, HCC1171, HCC1195, HCC15, HCC1588, HCC2108, HCC44	Forced-floating (not defined)
Pattni BS, 2016 [116]	Ovary (adenocarcinoma)	NCI/ADR-RES	Forced-floating (liquid overlay)
Perche F, 2012 [117]	Ovary (adenocarcinoma)	NCI/ADR-RES	Forced-floating (liquid overlay)
Preda P, 2023 [118]	Breast (adenocarcinoma), Brain (glioblastoma)	MDA-MB-231, U87	Scaffold-based methods
Pulze L, 2020 [119]	Breast (adenocarcinoma)	MCF-7	Forced-floating (ULA)
Raghavan S, 2016 [120]	Breast (adenocarcinoma), Ovary (adenocarcinoma)	MCF-7, OVCAR8	Hanging drop, Forced-floating (liquid overlay)
Raghavan S, 2019 [121]	Ovary (adenocarcinoma)	A2780, OVCAR3	Hanging drop
Ralph ACL, 2020 [122]	Breast (adenocarcinoma), Breast (carcinoma)	MCF-7, MDA-MB-231, T47D	Hanging drop
Roering P, 2022 [123]	Ovary (adenocarcinoma)	Patient-derived, CAOV3, OVCAR8	Forced-floating (ULA)
Roudi R, 2016 [124]	Lung (carcinoma)	A549	Forced-floating (poly-HEMA)
Rouhani M, 2014 [125]	Breast (carcinoma)	T47D	Forced-floating (liquid overlay)
Sakumoto M, 2018 [126]	Sarcoma	Patient-derived	Forced-floating (ULA)
Salehi F, 2020 [127]	Breast (adenocarcinoma), Breast (carcinoma)	MDA-MB-231, T47D, MCF-7	Forced-floating (liquid overlay), Hanging drop
Sambi M, 2020 [128]	Breast (adenocarcinoma)	MDA-MB-231	Scaffold-based methods
Sankar S, 2021 [129]	Lung (carcinoma)	A549	Recent advances
Särchen V, 2022 [130]	Sarcoma	RH30	Forced-floating (ULA)
Sarıyar E, 2023 [131]	Liver (carcinoma)	Huh7	Hanging drop
Sauer SJ, 2017 [132]	Breast (carcinoma), Breast (adenocarcinoma)	SUM149, SUM190, T47D, MCF-7	Forced-floating (ULA)
Shaheen S, 2016 [133]	Colon (carcinoma)	HCT-116	Forced-floating (not defined)
Shen K, 2014 [134]	Breast (adenocarcinoma)	MDA-MB-231	Scaffold-based methods
Sheth DB, 2019 [135]	Breast (adenocarcinoma)	MCF-7	Recent advances
Shortt RL, 2023 [136]	Colon (carcinoma)	HCT-116	Scaffold-based methods
Singh A, 2020 [137]	Breast (adenocarcinoma)	MCF-7	Scaffold-based methods
Suhito IR, 2021 [138]	Bone (neuroblastoma), Brain (glioblastoma)	SH-SY5Y, U-87	Recent advances
Tanenbaum LM, 2017 [139]	Ovary (adenocarcinoma)	UCI101, A2780	Forced-floating (not defined)
Tang S, 2017 [140]	Colon (adenocarcinoma), Ovary (adenocarcinoma)	HT-29, SKOV-3	Hanging drop
Taubenberger AV, 2019 [141]	Breast (adenocarcinoma)	MCF-7	Scaffold-based methods
Terrones M, 2024 [142]	Lung (adenocarcinoma)	HCC78	Forced-floating (ULA)
Tevis KM, 2017 [143]	Breast (adenocarcinoma)	MDA-MB-231	Scaffold-based methods
To HTN, 2022 [144]	Stomach (carcinoma)	SNU-216, SNU-484, SNU-601, SNU-638, SNU-668, and SNU-719	Forced-floating (ULA)
Torisawa YS, 2007 [145]	Breast (adenocarcinoma), Liver (carcinoma)	MCF-7, HepG2	Recent advances
Uematsu N, 2018 [146]	Breast (adenocarcinoma)	MCF-7	Recent advances
Varan G, 2021 [147]	Lung (carcinoma), Liver (carcinoma)	A549, HepG2	Forced-floating (poly-HEMA)
Vinci M, 2012 [148]	Brain (glioblastoma), Oral (carcinoma), Breast (adenocarcinoma)	U87, KNS42, LICR-LON-HN4, MDA-MB-231	Forced-floating (ULA), Agarose plates
Wan X, 2016 [149]	Colon (adenocarcinoma), Ovary (adenocarcinoma)	DLD-1, NCI/ADR	Scaffold-based methods
Wang Y, 2014 [150]	Cervix (adenocarcinoma)	HeLa	Scaffold-based methods
Ware MJ, 2016 [151]	Pancreas (carcinoma)	PANC-1, AsPc-1, BxPC-3, Capan-1, MIA PaCa-2 cells	Hanging drop
Wen Z, 2013 [152]	Pancreas (carcinoma)	MIAPaCa-2, PANC-1	Scaffold-based methods
Wenzel C, 2014 [153]	Breast (carcinoma)	T47D	Forced-floating (liquid overlay)
Weydert Z, 2020 [154]	Ovary (adenocarcinoma)	HEY, SKOV-3	Hanging drop
Wu G, 2019 [155]	Liver (carcinoma)	HepG2, Huh7	Scaffold-based methods
Wu KW, 2020 [156]	Bladder (carcinoma), Lung (carcinoma), Liver (carcinoma)	T24, A549, Huh-7	Recent advances
Xia H, 2020 [157]	Brain (glioblastoma)	LN229, U87	Scaffold-based methods
Xiong Q, 2023 [158]	Bladder	Patient-derived	Forced-floating (ULA)
Yamawaki K, 2021 [159]	Ovary	Patient-derived	Forced-floating (ULA)
Yoshida T, 2019 [160]	Bladder	Patient-derived	Scaffold-based methods
Yu L, 2015 [161]	Breast (adenocarcinoma)	MCF-7	Recent advances
Yu Q, 2021 [162]	Breast (adenocarcinoma)	MDA-MB-436, MDB-MB-231	Scaffold-based methods
Zhang JZ, 2012 [163]	Colon (adenocarcinoma), Ovary (teratocarcinoma)	DLD-1, PA-1 ovarian cancer cells	Forced-floating (liquid overlay)
Zhang JZ, 2012 [164]	Colon (adenocarcinoma)	DLD-1	Forced-floating (liquid overlay)
Zhang X, 2005 [165]	Breast (adenocarcinoma)	MCF-7	Recent advances
Zuchowska A, 2017 [166]	Liver (carcinoma)	HepG2	Recent advances

**Table 3:** Summary of the five most common spheroid sources.

Spheroid Formation Method	Number of Studies	Percentage of Total Number of Studies
Breast	46	24.6%
Colon	23	12.3%
Lung	20	10.7%
Ovary	18	9.6%
Brain	18	9.6%

**Table 4:** Summary of the five most common spheroid seeding methods.

Spheroid Formation Method	Number of Studies	Percentage of Total Number of Studies
Forced-floating	70	31.8%
Scaffold-based	41	18.6%
Recent advances	21	9.5%
Hanging drop	14	6.4%
Suspension culture	4	1.8%

**Table 5:** Summary of advantages and limitations of different seeding methods.

Method	Advantages	Limitations
Forced-floating method	Simple and scalable Produces uniform spheroids Compatible with automation Cost-effective	Spheroid integrity varies with cell density Limited nutrient and oxygen diffusion for large spheroids
Scaffold-based	Mimics ECM Supports long-term cultures Allows co-cultures Highly physiological	Variability in size and shape High cost of scaffolds Complexity in interpreting scaffold-induced effects
Hanging drop method	Low cost Produces uniform spheroids Minimal mechanical damage Control over spheroid size	Labor-intensive Limited scalability Potential damage during transfer Limited nutrient exchange
Suspension culture	Versatile Simple and cost-effective Suitable for high-throughput applications Closer mimicry of in vivo conditions	Nutrient and oxygen limitations in large spheroids Consistency challenges in terms of spheroid size
Microencapsulation	Provides controlled microenvironment Supports co-cultures Protects from shear stress	Nutrient diffusion limitations Challenges in retrieving spheroids
Bioprinting	Precise spheroid placement High control over architecture Effective for high-throughput applications	Requires optimization of bioinks Expensive equipment
Nanoparticle-assisted techniques	Enable precise aggregation Integrate with imaging/therapeutics Reproducible	Potential cytotoxicity of nanoparticles Scalability concerns
Microfluidics and lab-on-a-chip	Controlled environment Mimics in vivo gradients Real-time monitoring Supports co-cultures	High cost Requires technical expertise Limited scalability
Magnetic levitation	High control over size Supports co-cultures Possible to adapt to high-throughput applications Rapid and reproducible	Potential toxicity of nanoparticles Inconsistencies in particle uptake Equipment costs

## Data Availability

The original contributions presented in this study are included in the article. Further inquiries can be directed to the corresponding author.

## Abbreviations

The following abbreviations are used in this manuscript:

2D	Two-dimensional
3D	Three-dimensional
ECM	Extracellular matrix
NSCLC	Non-small-cell lung lymphoma
PEG	Polyethylene glycol
Poly-HEMA	Poly(2-hydroxyethyl methacrylate)
PLGA	Poly(lactic-co-glycolic) acid
SCLC	Small-cell lung cancer
ULA	Ultra-low attachment

## References

[1] Harrison R.G. (1910). The Outgrowth of the Nerve Fiber as a Mode of Protoplasmic Movement. J. Exp. Zool..

[2] Scherer W.F., Syverton J.T., Gey G.O. (1953). Studies on the Propagation in Vitro of Poliomyelitis Viruses. IV. Viral Multiplication in a Stable Strain of Human Malignant Epithelial Cells (Strain HeLa) Derived from an Epidermoid Carcinoma of the Cervix. J. Exp. Med..

[3] Cardoso B.D., Castanheira E.M.S., Lanceros-Méndez S., Cordoso V.F. (2023). Recent Advances on Cell Culture Platforms for In Vitro Drug Screening and Cell Therapies: From Conventional to Microfluidic Strategies. Adv. Healthc. Mater..

[4] Liu X., Raju P., Moo-Young M. (2011). 5.42—In Vitro Cancer Model for Drug Testing. Comprehensive Biotechnology.

[5] Kapałaczyńska M., Kolenda T., Przybyła W., Zajączkowska M., Teresiak A., Filas V., Ibbs M., Bliźniak R., Łuczewski Ł., Lamperska K. (2016). 2D and 3D Cell Cultures—A Comparison of Different Types of Cancer Cell Cultures. Arch. Med. Sci..

[6] Ravi M., Paramesh V., Kaviya S.R., Anuradha E., Solomon F.D.P. (2015). 3D Cell Culture Systems: Advantages and Applications. J. Cell. Physiol..

[7] Zanoni M., Piccinini F., Arienti C., Zamagni A., Santi S., Polico R., Bevilacqua A., Tesei A. (2016). 3D Tumor Spheroid Models for in Vitro Therapeutic Screening: A Systematic Approach to Enhance the Biological Relevance of Data Obtained. Sci. Rep..

[8] Ryoo H., Kimmel H., Rondo E., Underhill G.H. (2023). Advances in High Throughput Cell Culture Technologies for Therapeutic Screening and Biological Discovery Applications. Bioeng. Transl. Med..

[9] Hoarau-Véchot J., Rafii A., Touboul C., Pasquier J. (2018). Halfway between 2D and Animal Models: Are 3D Cultures the Ideal Tool to Study Cancer-Microenvironment Interactions?. Int. J. Mol. Sci..

[10] Breslin S., O’Driscoll L. (2016). The Relevance of Using 3D Cell Cultures, in Addition to 2D Monolayer Cultures, When Evaluating Breast Cancer Drug Sensitivity and Resistance. Oncotarget.

[11] Breslin S., O’Driscoll L. (2013). Three-Dimensional Cell Culture: The Missing Link in Drug Discovery. Drug Discov. Today.

[12] Jensen C., Teng Y. (2020). Is It Time to Start Transitioning From 2D to 3D Cell Culture?. Front. Mol. Biosci..

[13] Habanjar O., Diab-Assaf M., Caldefie-Chezet F., Delort L. (2021). 3D Cell Culture Systems: Tumor Application, Advantages, and Disadvantages. Int. J. Mol. Sci..

[14] Fontoura J.C., Viezzer C., dos Santos F.G., Ligabue R.A., Weinlich R., Puga R.D., Antonow D., Severino P., Bonorino C. (2020). Comparison of 2D and 3D Cell Culture Models for Cell Growth, Gene Expression and Drug Resistance. Mater. Sci. Eng. C.

[15] Abuwatfa W.H., Pitt W.G., Husseini G.A. (2024). Scaffold-Based 3D Cell Culture Models in Cancer Research. J. Biomed. Sci..

[16] Franchi-Mendes T., Eduardo R., Domenici G., Brito C. (2021). 3D Cancer Models: Depicting Cellular Crosstalk within the Tumour Microenvironment. Cancers.

[17] Park C.H., Park J.H., Suh Y.J. (2024). Perspective of 3D Culture in Medicine: Transforming Disease Research and Therapeutic Applications. Front. Bioeng. Biotechnol..

[18] (2003). Goodbye, flat biology?. Nature.

[19] Yu Y., Zhao Y., Zou Y., Lu C., Li N., Shi Z., Li X., Lai X. (2025). Ultra-Sensitive pH Responsive Hydrogels with Injectable and Self-Healing Performance for Controlled Drug Delivery. Int. J. Pharm. X.

[20] Sutherland R.M., McCredie J.A., Inch W.R. (1971). Growth of Multicell Spheroids in Tissue Culture as a Model of Nodular Carcinomas. JNCI J. Natl. Cancer Inst..

[21] El Harane S., Zidi B., El Harane N., Krause K.-H., Matthes T., Preynat-Seauve O. (2023). Cancer Spheroids and Organoids as Novel Tools for Research and Therapy: State of the Art and Challenges to Guide Precision Medicine. Cells.

[22] Gilazieva Z., Ponomarev A., Rutland C., Rizvanov A., Solovyeva V. (2020). Promising Applications of Tumor Spheroids and Organoids for Personalized Medicine. Cancers.

[23] Nayak P., Bentivoglio V., Varani M., Signore A. (2023). Three-Dimensional In Vitro Tumor Spheroid Models for Evaluation of Anticancer Therapy: Recent Updates. Cancers.

[24] Agastin S., Giang U.-B.T., Geng Y., DeLouise L.A., King M.R. (2011). Continuously Perfused Microbubble Array for 3D Tumor Spheroid Model. Biomicrofluidics.

[25] Alhasan L., Qi A., Al-Abboodi A., Rezk A., Chan P.P.Y., Iliescu C., Yeo L.Y. (2016). Rapid Enhancement of Cellular Spheroid Assembly by Acoustically Driven Microcentrifugation. ACS Biomater. Sci. Eng..

[26] An H.J., Kim H.S., Kwon J.A., Song J., Choi I. (2020). Adjustable and Versatile 3D Tumor Spheroid Culture Platform with Interfacial Elastomeric Wells. ACS Appl. Mater. Interfaces.

[27] Árnadóttir S.S., Jeppesen M., Lamy P., Bramsen J.B., Nordentoft I., Knudsen M., Vang S., Madsen M.R., Thastrup O., Thastrup J. (2018). Characterization of Genetic Intratumor Heterogeneity in Colorectal Cancer and Matching Patient-Derived Spheroid Cultures. Mol. Oncol..

[28] Baek N., Seo O.W., Lee J., Hulme J., An S.S.A. (2016). Real-Time Monitoring of Cisplatin Cytotoxicity on Three-Dimensional Spheroid Tumor Cells. Drug Des. Dev. Ther..

[29] Barone R.M., Calabro-Jones P., Thomas T.N., Sharp T.R., Byfield J.E. (1981). Surgical Adjuvant Therapy in Colon Carcinoma: A Human Tumor Spheroid Model for Evaluating Radiation Sensitizing Agents. Cancer.

[30] Bartholomä P., Impidjati, Reininger-Mack A., Zhang Z., Thielecke H., Robitzki A. (2005). A More Aggressive Breast Cancer Spheroid Model Coupled to an Electronic Capillary Sensor System for a High-Content Screening of Cytotoxic Agents in Cancer Therapy: 3-Dimensional in Vitro Tumor Spheroids as a Screening Model. J. Biomol. Screen..

[31] Boo L., Yeap S.K., Ali N.M., Ho W.Y., Ky H., Satharasinghe D.A., Liew W.C., Tan S.W., Wang M.-L., Cheong S.K. (2020). Phenotypic and microRNA Characterization of the Neglected CD24+ Cell Population in MCF-7 Breast Cancer 3-Dimensional Spheroid Culture. J. Chin. Med. Assoc..

[32] Brooks E.A., Gencoglu M.F., Corbett D.C., Stevens K.R., Peyton S.R. (2019). An Omentum-Inspired 3D PEG Hydrogel for Identifying ECM-Drivers of Drug Resistant Ovarian Cancer. APL Bioeng..

[33] Bruns J., Egan T., Mercier P., Zustiak S.P. (2023). Glioblastoma Spheroid Growth and Chemotherapeutic Responses in Single and Dual-Stiffness Hydrogels. Mech. Cells Fibers.

[34] Calori I.R., Alves S.R., Bi H., Tedesco A.C. (2022). Type-I Collagen/Collagenase Modulates the 3D Structure and Behavior of Glioblastoma Spheroid Models. ACS Appl. Bio Mater..

[35] Chang S., Wen J., Su Y., Ma H. (2022). Microfluidic Platform for Studying the Anti-Cancer Effect of Ursolic Acid on Tumor Spheroid. Electrophoresis.

[36] Chen G., Liu W., Yan B. (2022). Breast Cancer MCF-7 Cell Spheroid Culture for Drug Discovery and Development. J. Cancer Ther..

[37] Chen M.C.W., Gupta M., Cheung K.C. (2010). Alginate-Based Microfluidic System for Tumor Spheroid Formation and Anticancer Agent Screening. Biomed. Microdevices.

[38] Chen Z., Ma N., Sun X., Li Q., Zeng Y., Chen F., Sun S., Xu J., Zhang J., Ye H. (2021). Automated Evaluation of Tumor Spheroid Behavior in 3D Culture Using Deep Learning-Based Recognition. Biomaterials.

[39] Cheng V., Esteves F., Chakrabarty A., Cockle J., Short S., Brüning-Richardson A. (2015). High-Content Analysis of Tumour Cell Invasion in Three-Dimensional Spheroid Assays. Oncoscience.

[40] Close D.A., Johnston P.A. (2022). Detection and Impact of Hypoxic Regions in Multicellular Tumor Spheroid Cultures Formed by Head and Neck Squamous Cell Carcinoma Cells Lines. SLAS Discov..

[41] Das T., Meunier L., Barbe L., Provencher D., Guenat O., Gervais T., Mes-Masson A.-M. (2013). Empirical Chemosensitivity Testing in a Spheroid Model of Ovarian Cancer Using a Microfluidics-Based Multiplex Platform. Biomicrofluidics.

[42] Das V., Fürst T., Gurská S., Džubák P., Hajdúch M. (2016). Reproducibility of Uniform Spheroid Formation in 384-Well Plates: The Effect of Medium Evaporation. SLAS Discov..

[43] Das V., Fürst T., Gurská S., Džubák P., Hajdúch M. (2017). Evaporation-Reducing Culture Condition Increases the Reproducibility of Multicellular Spheroid Formation in Microtiter Plates. J. Vis. Exp..

[44] De Angelis M.L., Bruselles A., Francescangeli F., Pucilli F., Vitale S., Zeuner A., Tartaglia M., Baiocchi M. (2018). Colorectal Cancer Spheroid Biobanks: Multi-Level Approaches to Drug Sensitivity Studies. Cell Biol. Toxicol..

[45] Dhamecha D., Le D., Chakravarty T., Perera K., Dutta A., Menon J.U. (2021). Fabrication of PNIPAm-Based Thermoresponsive Hydrogel Microwell Arrays for Tumor Spheroid Formation. Mater. Sci. Eng. C.

[46] Dias D.R., Moreira A.F., Correia I.J. (2016). The Effect of the Shape of Gold Core–Mesoporous Silica Shell Nanoparticles on the Cellular Behavior and Tumor Spheroid Penetration. J. Mater. Chem. B.

[47] Domenici G., Eduardo R., Castillo-Ecija H., Orive G., Montero Carcaboso Á., Brito C. (2021). PDX-Derived Ewing’s Sarcoma Cells Retain High Viability and Disease Phenotype in Alginate Encapsulated Spheroid Cultures. Cancers.

[48] Dufau I., Frongia C., Sicard F., Dedieu L., Cordelier P., Ausseil F., Ducommun B., Valette A. (2012). Multicellular Tumor Spheroid Model to Evaluate Spatio-Temporal Dynamics Effect of Chemotherapeutics: Application to the Gemcitabine/CHK1 Inhibitor Combination in Pancreatic Cancer. BMC Cancer.

[49] Eetezadi S., Evans J.C., Shen Y.-T., de Souza R., Piquette-Miller M., Allen C. (2017). Ratio-Dependent Synergism of a Doxorubicin and Olaparib Combination in 2D and Spheroid Models of Ovarian Cancer. Mol. Pharm..

[50] Eguchi H., Kimura R., Onuma S., Ito A., Yu Y., Yoshino Y., Matsunaga T., Endo S., Ikari A. (2022). Elevation of Anticancer Drug Toxicity by Caffeine in Spheroid Model of Human Lung Adenocarcinoma A549 Cells Mediated by Reduction in Claudin-2 and Nrf2 Expression. Int. J. Mol. Sci..

[51] Eimer S., Dugay F., Airiau K., Avril T., Quillien V., Belaud-Rotureau M.-A., Belloc F. (2012). Cyclopamine Cooperates with EGFR Inhibition to Deplete Stem-like Cancer Cells in Glioblastoma-Derived Spheroid Cultures. Neuro-Oncology.

[52] El-Sadek I.A., Miyazawa A., Shen L.T.-W., Makita S., Mukherjee P., Lichtenegger A., Matsusaka S., Yasuno Y. (2021). Three-Dimensional Dynamics Optical Coherence Tomography for Tumor Spheroid Evaluation. Biomed. Opt. Express.

[53] Enmon R.M., O’Connor K.C., Lacks D.J., Schwartz D.K., Dotson R.S. (2001). Dynamics of Spheroid Self-Assembly in Liquid-Overlay Culture of DU 145 Human Prostate Cancer Cells. Biotechnol. Bioeng..

[54] Flørenes V.A., Flem-Karlsen K., McFadden E., Bergheim I.R., Nygaard V., Nygård V., Farstad I.N., Øy G.F., Emilsen E., Giller-Fleten K. (2019). A Three-Dimensional Ex Vivo Viability Assay Reveals a Strong Correlation Between Response to Targeted Inhibitors and Mutation Status in Melanoma Lymph Node Metastases. Transl. Oncol..

[55] Fu J., Li X.B., Wang L.X., Lv X.H., Lu Z., Wang F., Xia Q., Yu L., Li C.M. (2020). One-Step Dip-Coating-Fabricated Core–Shell Silk Fibroin Rice Paper Fibrous Scaffolds for 3D Tumor Spheroid Formation. ACS Appl. Bio Mater..

[56] Fu J.J., Zhou Y., Shi X.X., Kang Y.J., Lu Z.S., Li Y., Li C.M., Yu L. (2019). Spontaneous Formation of Tumor Spheroid on a Hydrophilic Filter Paper for Cancer Stem Cell Enrichment. Colloids Surf. B Biointerfaces.

[57] Gao Y., Wu M., Luan Q., Papautsky I., Xu J. (2022). Acoustic Bubble for Spheroid Trapping, Rotation, and Culture: A Tumor-on-a-Chip Platform (ABSTRACT Platform). Lab Chip.

[58] Gencoglu M.F., Barney L.E., Hall C.L., Brooks E.A., Peyton S.R. (2018). Comparative Study of Multicellular Tumor Spheroid Formation Methods and Implications for Drug Screening. ACS Biomater. Sci. Eng..

[59] Gendre D.A.J., Ameti E., Karenovics W., Perriraz-Mayer N., Triponez F., Serre-Beinier V. (2021). Optimization of Tumor Spheroid Model in Mesothelioma and Lung Cancers and Anti-Cancer Drug Testing in H2052/484 Spheroids. Oncotarget.

[60] Gheytanchi E., Naseri M., Karimi-Busheri F., Atyabi F., Mirsharif E.S., Bozorgmehr M., Ghods R., Madjd Z. (2021). Morphological and Molecular Characteristics of Spheroid Formation in HT-29 and Caco-2 Colorectal Cancer Cell Lines. Cancer Cell Int..

[61] Goisnard A., Daumar P., Dubois C., Aubel C., Roux M., Depresle M., Gauthier J., Vidalinc B., Penault-Llorca F., Mounetou E. (2021). LightSpot^®^-FL-1 Fluorescent Probe: An Innovative Tool for Cancer Drug Resistance Analysis by Direct Detection and Quantification of the P-glycoprotein (P-gp) on Monolayer Culture and Spheroid Triple Negative Breast Cancer Models. Cancers.

[62] Guo X., Chen Y., Ji W., Chen X., Li C., Ge R. (2019). Enrichment of Cancer Stem Cells by Agarose Multi-Well Dishes and 3D Spheroid Culture. Cell Tissue Res..

[63] Hagemann J., Jacobi C., Hahn M., Schmid V., Welz C., Schwenk-Zieger-ZIEGER S., Stauber R., Baumeister P., Becker S. (2017). Spheroid-Based 3D Cell Cultures Enable Personalized Therapy Testing and Drug Discovery in Head and Neck Cancer. Anticancer Res..

[64] Han S., Lim J.Y., Cho K., Lee H.W., Park J.Y., Ro S.W., Kim K.S., Seo H.R., Kim D.Y. (2022). Anti-Cancer Effects of YAP Inhibitor (CA3) in Combination with Sorafenib against Hepatocellular Carcinoma (HCC) in Patient-Derived Multicellular Tumor Spheroid Models (MCTS). Cancers.

[65] Harmer J., Struve N., Brüning-Richardson A. (2019). Characterization of the Effects of Migrastatic Inhibitors on 3D Tumor Spheroid Invasion by High-Resolution Confocal Microscopy. J. Vis. Exp..

[66] Herter S., Morra L., Schlenker R., Sulcova J., Fahrni L., Waldhauer I., Lehmann S., Reisländer T., Agarkova I., Kelm J.M. (2017). A Novel Three-Dimensional Heterotypic Spheroid Model for the Assessment of the Activity of Cancer Immunotherapy Agents. Cancer Immunol. Immunother..

[67] Ho W.Y., Yeap S.K., Ho C.L., Rahim R.A., Alitheen N.B. (2012). Development of Multicellular Tumor Spheroid (MCTS) Culture from Breast Cancer Cell and a High Throughput Screening Method Using the MTT Assay. PLoS ONE.

[68] Ho W.Y., Liew S.S., Yeap S.K., Alitheen N.B. (2021). Synergistic Cytotoxicity between Elephantopus Scaber and Tamoxifen on MCF-7-Derived Multicellular Tumor Spheroid. Evid. -Based Complement. Altern. Med..

[69] Hofmann S., Cohen-Harazi R., Maizels Y., Koman I. (2021). Patient-Derived Tumor Spheroid Cultures as a Promising Tool to Assist Personalized Therapeutic Decisions in Breast Cancer. Transl. Cancer Res..

[70] Hornung A., Poettler M., Friedrich R.P., Weigel B., Duerr S., Zaloga J., Cicha I., Alexiou C., Janko C. (2016). Toxicity of Mitoxantrone-Loaded Superparamagnetic Iron Oxide Nanoparticles in a HT-29 Tumour Spheroid Model. Anticancer Res..

[71] Huang Z., Yu P., Tang J. (2020). Characterization of Triple-Negative Breast Cancer MDA-MB-231 Cell Spheroid Model. OncoTargets Ther..

[72] Jove M., Spencer J.A., Hubbard M.E., Holden E.C., O’Dea R.D., Brook B.S., Phillips R.M., Smye S.W., Loadman P.M., Twelves C.J. (2019). Cellular Uptake and Efflux of Palbociclib In Vitro in Single Cell and Spheroid Models. J. Pharmacol. Exp. Ther..

[73] Ju F.N., Kim C.-H., Lee K.-H., Kim C.-D., Lim J., Lee T., Park C.G., Kim T.-H. (2023). Gold Nanostructure-Integrated Conductive Microwell Arrays for Uniform Cancer Spheroid Formation and Electrochemical Drug Screening. Biosens. Bioelectron..

[74] Karamikamkar S., Behzadfar E., Cheung K.C. (2018). A Novel Approach to Producing Uniform 3-D Tumor Spheroid Constructs Using Ultrasound Treatment. Biomed. Microdevices.

[75] Karlsson H., Fryknäs M., Larsson R., Nygren P. (2012). Loss of Cancer Drug Activity in Colon Cancer HCT-116 Cells during Spheroid Formation in a New 3-D Spheroid Cell Culture System. Exp. Cell Res..

[76] Karshieva S.S., Glinskaya E.G., Dalina A.A., Akhlyustina E.V., Makarova E.A., Khesuani Y.D., Chmelyuk N.S., Abakumov M.A., Khochenkov D.A., Mironov V.A. (2022). Antitumor Activity of Photodynamic Therapy with Tetracationic Derivative of Synthetic Bacteriochlorin in Spheroid Culture of Liver and Colon Cancer Cells. Photodiagn. Photodyn. Ther..

[77] Kato E.E., Sampaio S.C. (2021). Crotoxin Modulates Events Involved in Epithelial–Mesenchymal Transition in 3D Spheroid Model. Toxins.

[78] Kim C.-H., Suhito I.R., Angeline N., Han Y., Son H., Luo Z., Kim T.-H. (2020). Vertically Coated Graphene Oxide Micro-Well Arrays for Highly Efficient Cancer Spheroid Formation and Drug Screening. Adv. Healthc. Mater..

[79] Ko J., Ahn J., Kim S., Lee Y., Lee J., Park D., Jeon N.L. (2019). Tumor Spheroid-on-a-Chip: A Standardized Microfluidic Culture Platform for Investigating Tumor Angiogenesis. Lab Chip.

[80] Kochanek S.J., Close D.A., Johnston P.A. (2019). High Content Screening Characterization of Head and Neck Squamous Cell Carcinoma Multicellular Tumor Spheroid Cultures Generated in 384-Well Ultra-Low Attachment Plates to Screen for Better Cancer Drug Leads. Assay Drug Dev. Technol..

[81] Kochanek S.J., Close D.A., Camarco D.P., Johnston P.A. (2020). Maximizing the Value of Cancer Drug Screening in Multicellular Tumor Spheroid Cultures: A Case Study in Five Head and Neck Squamous Cell Carcinoma Cell Lines. SLAS Discov..

[82] Koshkin V., Ailles L.E., Liu G., Krylov S.N. (2016). Metabolic Suppression of a Drug-Resistant Subpopulation in Cancer Spheroid Cells. J. Cell. Biochem..

[83] Kroupová J., Hanuš J., Štěpánek F. (2022). Surprising Efficacy Twist of Two Established Cytostatics Revealed by A-La-Carte 3D Cell Spheroid Preparation Protocol. Eur. J. Pharm. Biopharm..

[84] Kudláčová J., Kotrchová L., Kostka L., Randárová E., Filipová M., Janoušková O., Fang J., Etrych T. (2020). Structure-to-Efficacy Relationship of HPMA-Based Nanomedicines: The Tumor Spheroid Penetration Study. Pharmaceutics.

[85] Kumari P., Jain S., Ghosh B., Zorin V., Biswas S. (2017). Polylactide-Based Block Copolymeric Micelles Loaded with Chlorin E6 for Photodynamic Therapy: In Vitro Evaluation in Monolayer and 3D Spheroid Models. Mol. Pharm..

[86] Lal-Nag M., McGee L., Titus S.A., Brimacombe K., Michael S., Sittampalam G., Ferrer M. (2017). Exploring Drug Dosing Regimens In Vitro Using Real-Time 3D Spheroid Tumor Growth Assays. SLAS Discov..

[87] Lama R., Zhang L., Naim J.M., Williams J., Zhou A., Su B. (2013). Development, Validation and Pilot Screening of an in Vitro Multi-Cellular Three-Dimensional Cancer Spheroid Assay for Anti-Cancer Drug testing. Bioorg. Med. Chem..

[88] Landgraf L., Kozlowski A., Zhang X., Fournelle M., Becker F.-J., Tretbar S., Melzer A. (2022). Focused Ultrasound Treatment of a Spheroid In Vitro Tumour Model. Cells.

[89] Le V.M., Lang M.-D., Shi W.-B., Liu J.-W. (2016). A Collagen-Based Multicellular Tumor Spheroid Model for Evaluation of the Efficiency of Nanoparticle Drug Delivery. Artif. Cells Nanomed. Biotechnol..

[90] Lee S.W., Hong S., Jung B., Jeong S.Y., Byeon J.H., Jeong G.S., Choi J., Hwang C. (2019). In Vitro Lung Cancer Multicellular Tumor Spheroid Formation Using a Microfluidic Device. Biotechnol. Bioeng..

[91] Lee Y., Chen Z., Lim W., Cho H., Park S. (2022). High-Throughput Screening of Anti-Cancer Drugs Using a Microfluidic Spheroid Culture Device with a Concentration Gradient Generator. Curr. Protoc..

[92] Lemmo S., Atefi E., Luker G.D., Tavana H. (2014). Optimization of Aqueous Biphasic Tumor Spheroid Microtechnology for Anti-Cancer Drug Testing in 3D Culture. Cell. Mol. Bioeng..

[93] Li M., Lu B., Dong X., Zhou Y., He Y., Zhang T., Bao L. (2019). Enhancement of Cisplatin-Induced Cytotoxicity against Cervical Cancer Spheroid Cells by Targeting Long Non-Coding RNAs. Pathol. Res. Pract..

[94] Lim W., Park S. (2018). A Microfluidic Spheroid Culture Device with a Concentration Gradient Generator for High-Throughput Screening of Drug Efficacy. Molecules.

[95] Lin Z.-T., Gu J., Wang H., Wu A., Sun J., Chen S., Li Y., Kong Y., Wu M.X., Wu T. (2021). Thermosensitive and Conductive Hybrid Polymer for Real-Time Monitoring of Spheroid Growth and Drug Responses. ACS Sens..

[96] Liu X., Lin H., Song J., Zhang T., Wang X., Huang X., Zheng C. (2021). A Novel SimpleDrop Chip for 3D Spheroid Formation and Anti-Cancer Drug Assay. Micromachines.

[97] Lorenzo C., Frongia C., Jorand R., Fehrenbach J., Weiss P., Maandhui A., Gay G., Ducommun B., Lobjois V. (2011). Live Cell Division Dynamics Monitoring in 3D Large Spheroid Tumor Models Using Light Sheet Microscopy. Cell Div..

[98] Luan Q., Becker J.H., Macaraniag C., Massad M.G., Zhou J., Shimamura T., Papautsky I. (2022). Non-Small Cell Lung Carcinoma Spheroid Models in Agarose Microwells for Drug Response Studies. Lab Chip.

[99] Madsen N.H., Nielsen B.S., Nhat S.L., Skov S., Gad M., Larsen J. (2021). Monocyte Infiltration and Differentiation in 3D Multicellular Spheroid Cancer Models. Pathogens.

[100] Marshall S.K., Saelim B., Taweesap M., Pachana V., Panrak Y., Makchuchit N., Jaroenpakdee P. (2022). Anti-EGFR Targeted Multifunctional I-131 Radio-Nanotherapeutic for Treating Osteosarcoma: In Vitro 3D Tumor Spheroid Model. Nanomaterials.

[101] Maruhashi R., Akizuki R., Sato T., Matsunaga T., Endo S., Yamaguchi M., Yamazaki Y., Sakai H., Ikari A. (2018). Elevation of Sensitivity to Anticancer Agents of Human Lung Adenocarcinoma A549 Cells by Knockdown of Claudin-2 Expression in Monolayer and Spheroid Culture Models. Biochim. Biophys. Acta Mol. Cell Res..

[102] Melnik D., Sahana J., Corydon T.J., Kopp S., Nassef M.Z., Wehland M., Infanger M., Grimm D., Krüger M. (2020). Dexamethasone Inhibits Spheroid Formation of Thyroid Cancer Cells Exposed to Simulated Microgravity. Cells.

[103] Molyneaux K., Wnek M.D., Craig S.E.L., Vincent J., Rucker I., Wnek G.E., Brady-Kalnay S.M. (2021). Physically-Cross-Linked Poly(Vinyl Alcohol) Cell Culture Plate Coatings Facilitate Preservation of Cell–Cell Interactions, Spheroid Formation, and Stemness. J. Biomed. Mater. Res. B Appl. Biomater..

[104] Monazzam A., Josephsson R., Blomqvist C., Carlsson J., Långström B., Bergström M. (2007). Application of the Multicellular Tumour Spheroid Model to Screen PET Tracers for Analysis of Early Response of Chemotherapy in Breast Cancer. Breast Cancer Res..

[105] Morimoto T., Takemura Y., Miura T., Yamamoto T., Kakizaki F., An H., Maekawa H., Yamaura T., Kawada K., Sakai Y. (2023). Novel and Efficient Method for Culturing Patient-Derived Gastric Cancer Stem Cells. Cancer Sci..

[106] Mosaad E.O., Chambers K.F., Futrega K., Clements J.A., Doran M.R. (2018). The Microwell-Mesh: A High-Throughput 3D Prostate Cancer Spheroid and Drug-Testing Platform. Sci. Rep..

[107] Mueggler A., Pilotto E., Perriraz-Mayer N., Jiang S., Addeo A., Bédat B., Karenovics W., Triponez F., Serre-Beinier V. (2023). An Optimized Method to Culture Human Primary Lung Tumor Cell Spheroids. Cancers.

[108] Nashimoto Y., Okada R., Hanada S., Arima Y., Nishiyama K., Miura T., Yokokawa R. (2020). Vascularized Cancer on a Chip: The Effect of Perfusion on Growth and Drug Delivery of Tumor Spheroid. Biomaterials.

[109] Nigjeh S.E., Yeap S.K., Nordin N., Kamalideghan B., Ky H., Rosli R. (2018). Citral Induced Apoptosis in MDA-MB-231 Spheroid Cells. BMC Complement. Altern. Med..

[110] Nittayaboon K., Leetanaporn K., Sangkhathat S., Roytrakul S., Navakanitworakul R. (2022). Cytotoxic Effect of Metformin on Butyrate-Resistant PMF-K014 Colorectal Cancer Spheroid cells. Biomed. Pharmacother..

[111] Ohya S., Kajikuri J., Endo K., Kito H., Matsui M. (2021). KCa1.1 K+ Channel Inhibition Overcomes Resistance to Antiandrogens and Doxorubicin in a Human Prostate Cancer LNCaP Spheroid Model. Int. J. Mol. Sci..

[112] Oliveira M.S., Aryasomayajula B., Pattni B., Mussi S.V., Ferreira L.A.M., Torchilin V.P. (2016). Solid Lipid Nanoparticles Co-Loaded with Doxorubicin and α-Tocopherol Succinate Are Effective against Drug-Resistant Cancer Cells in Monolayer and 3-D Spheroid Cancer Cell Models. Int. J. Pharm..

[113] Ono K., Sato K., Nakamura T., Yoshida Y., Murata S., Yoshida K., Kanemoto H., Umemori K., Kawai H., Obata K. (2022). Reproduction of the Antitumor Effect of Cisplatin and Cetuximab Using a Three-Dimensional Spheroid Model in Oral Cancer. Int. J. Med. Sci..

[114] Pampaloni F., Mayer B., Kabat Vel-Job K., Ansari N., Hötte K., Kögel D., Stelzer E.H.K. (2017). A Novel Cellular Spheroid-Based Autophagy Screen Applying Live Fluorescence Microscopy Identifies Nonactin as a Strong Inducer of Autophagosomal Turnover. SLAS Discov..

[115] Park M.C., Jeong H., Son S.H., Kim Y., Han D., Goughnour P.C., Kang T., Kwon N.H., Moon H.E., Paek S.H. (2016). Novel Morphologic and Genetic Analysis of Cancer Cells in a 3D Microenvironment Identifies STAT3 as a Regulator of Tumor Permeability Barrier Function. Cancer Res..

[116] Pattni B.S., Nagelli S.G., Aryasomayajula B., Deshpande P.P., Kulkarni A., Hartner W.C., Thakur G., Degterev A., Torchilin V.P. (2016). Targeting of Micelles and Liposomes Loaded with the Pro-Apoptotic Drug, NCL-240, into NCI/ADR-RES Cells in a 3D Spheroid Model. Pharm. Res..

[117] Perche F., Patel N.R., Torchilin V.P. (2012). Accumulation and Toxicity of Antibody-Targeted Doxorubicin-Loaded PEG–PE Micelles in Ovarian Cancer Cell Spheroid Model. J. Control. Release.

[118] Preda P., Enciu A.-M., Tanase C., Dudau M., Albulescu L., Maxim M.-E., Darie-Niță R.N., Brincoveanu O., Avram M. (2023). Assessing Polysaccharides/Aloe Vera–Based Hydrogels for Tumor Spheroid Formation. Gels.

[119] Pulze L., Congiu T., Brevini T.A.L., Grimaldi A., Tettamanti G., D’Antona P., Baranzini N., Acquati F., Ferraro F., de Eguileor M. (2020). MCF7 Spheroid Development: New Insight about Spatio/Temporal Arrangements of TNTs, Amyloid Fibrils, Cell Connections, and Cellular Bridges. Int. J. Mol. Sci..

[120] Raghavan S., Mehta P., Horst E.N., Ward M.R., Rowley K.R., Mehta G. (2016). Comparative Analysis of Tumor Spheroid Generation Techniques for Differential in Vitro Drug Toxicity. Oncotarget.

[121] Raghavan S., Mehta P., Xie Y., Lei Y.L., Mehta G. (2019). Ovarian Cancer Stem Cells and Macrophages Reciprocally Interact through the WNT Pathway to Promote Pro-Tumoral and Malignant Phenotypes in 3D Engineered Microenvironments. J. Immunother. Cancer.

[122] Ralph A.C.L., Valadão I.C., Cardoso E.C., Martins V.R., Oliveira L.M.S., Bevilacqua E.M.A.F., Geraldo M.V., Jaeger R.G., Goldberg G.S., Freitas V.M. (2020). Environmental Control of Mammary Carcinoma Cell Expansion by Acidification and Spheroid Formation in Vitro. Sci. Rep..

[123] Roering P., Siddiqui A., Heuser V.D., Potdar S., Mikkonen P., Oikkonen J., Li Y., Pikkusaari S., Wennerberg K., Hynninen J. (2022). Effects of Wee1 Inhibitor Adavosertib on Patient-Derived High-Grade Serous Ovarian Cancer Cells Are Multiple and Independent of Homologous Recombination Status. Front. Oncol..

[124] Roudi R., Madjd Z., Ebrahimi M., Najafi A., Korourian A., Shariftabrizi A., Samadikuchaksaraei A. (2016). Evidence for Embryonic Stem-like Signature and Epithelial-Mesenchymal Transition Features in the Spheroid Cells Derived from Lung Adenocarcinoma. Tumor Biol..

[125] Rouhani M., Goliaei B., Khodagholi F., Nikoofar A. (2014). Lithium Increases Radiosensitivity by Abrogating DNA Repair in Breast Cancer Spheroid Culture. Arch. Iran. Med..

[126] Sakumoto M., Oyama R., Takahashi M., Takai Y., Kito F., Shiozawa K., Qiao Z., Endo M., Yoshida A., Kawai A. (2018). Establishment and Proteomic Characterization of Patient-Derived Clear Cell Sarcoma Xenografts and Cell Lines. In Vitro Cell. Dev. Biol. Anim..

[127] Salehi F., Jamali T., Kavoosi G., Ardestani S.K., Vahdati S.N. (2020). Stabilization of Zataria Essential Oil with Pectin-Based Nanoemulsion for Enhanced Cytotoxicity in Monolayer and Spheroid Drug-Resistant Breast Cancer Cell Cultures and Deciphering Its Binding Mode with gDNA. Int. J. Biol. Macromol..

[128] Sambi M., Samuel V., Qorri B., Haq S., Burov S.V., Markvicheva E., Harless W., Szewczuk M.R. (2020). A Triple Combination of Metformin, Acetylsalicylic Acid, and Oseltamivir Phosphate Impacts Tumour Spheroid Viability and Upends Chemoresistance in Triple-Negative Breast Cancer. Drug Des. Dev. Ther..

[129] Sankar S., Mehta V., Ravi S., Sharma C.S., Rath S.N. (2021). A Novel Design of Microfluidic Platform for Metronomic Combinatorial Chemotherapy Drug Screening Based on 3D Tumor Spheroid Model. Biomed. Microdevices.

[130] Särchen V., Shanmugalingam S., Kehr S., Reindl L.M., Greze V., Wiedemann S., Boedicker C., Jacob M., Bankov K., Becker N. (2022). Pediatric Multicellular Tumor Spheroid Models Illustrate a Therapeutic Potential by Combining BH3 Mimetics with Natural Killer (NK) Cell-Based Immunotherapy. Cell Death Discov..

[131] Sarıyar E., Karpat O., Sezan S., Baylan S.M., Kıpçak A., Guven K., Erdal E., Fırtına Karagonlar Z. (2023). EGFR and Lyn Inhibition Augments Regorafenib Induced Cell Death in Sorafenib Resistant 3D Tumor Spheroid Model. Cell. Signal..

[132] Sauer S.J., Tarpley M., Shah I., Save A.V., Lyerly H.K., Patierno S.R., Williams K.P., Devi G.R. (2017). Bisphenol A Activates EGFR and ERK Promoting Proliferation, Tumor Spheroid Formation and Resistance to EGFR Pathway Inhibition in Estrogen Receptor-Negative Inflammatory Breast Cancer Cells. Carcinogenesis.

[133] Shaheen S., Ahmed M., Lorenzi F., Nateri A.S. (2016). Spheroid-Formation (Colonosphere) Assay for in Vitro Assessment and Expansion of Stem Cells in Colon Cancer. Stem Cell Rev. Rep..

[134] Shen K., Lee J., Yarmush M.L., Parekkadan B. (2014). Microcavity Substrates Casted from Self-Assembled Microsphere Monolayers for Spheroid Cell Culture. Biomed. Microdevices.

[135] Sheth D.B., Gratzl M. (2019). Electrochemical Mapping of Oxygenation in the Three-Dimensional Multicellular Tumour Hemi-Spheroid. Proc. Math. Phys. Eng. Sci..

[136] Shortt R.L., Wang Y., Hummon A.B., Jones L.M. (2023). Development of Spheroid-FPOP: An In-Cell Protein Footprinting Method for 3D Tumor Spheroids. J. Am. Soc. Mass Spectrom..

[137] Singh A., Tayalia P. (2020). Three-Dimensional Cryogel Matrix for Spheroid Formation and Anti-Cancer Drug Screening. J. Biomed. Mater. Res. A.

[138] Suhito I.R., Angeline N., Lee K.-H., Kim H., Park C.G., Luo Z., Kim T.-H. (2021). A Spheroid-Forming Hybrid Gold Nanostructure Platform That Electrochemically Detects Anticancer Effects of Curcumin in a Multicellular Brain Cancer Model. Small.

[139] Tanenbaum L.M., Mantzavinou A., Subramanyam K.S., del Carmen M.G., Cima M.J. (2017). Ovarian Cancer Spheroid Shrinkage Following Continuous Exposure to Cisplatin Is a Function of Spheroid Diameter. Gynecol. Oncol..

[140] Tang S., Hu K., Sun J., Li Y., Guo Z., Liu M., Liu Q., Zhang F., Gu N. (2017). High Quality Multicellular Tumor Spheroid Induction Platform Based on Anisotropic Magnetic Hydrogel. ACS Appl. Mater. Interfaces.

[141] Taubenberger A.V., Girardo S., Träber N., Fischer-Friedrich E., Kräter M., Wagner K., Kurth T., Richter I., Haller B., Binner M. (2019). 3D Microenvironment Stiffness Regulates Tumor Spheroid Growth and Mechanics via P21 and ROCK. Adv. Biosyst..

[142] Terrones M., Deben C., Rodrigues-Fortes F., Schepers A., de Beeck K.O., Van Camp G., Vandeweyer G. (2024). CRISPR/Cas9-Edited ROS1 + Non-Small Cell Lung Cancer Cell Lines Highlight Differential Drug Sensitivity in 2D vs. 3D Cultures While Reflecting Established Resistance Profiles. J. Transl. Med..

[143] Tevis K.M., Cecchi R.J., Colson Y.L., Grinstaff M.W. (2017). Mimicking the Tumor Microenvironment to Regulate Macrophage Phenotype and Assessing Chemotherapeutic Efficacy in Embedded Cancer Cell/Macrophage Spheroid Models. Acta Biomater..

[144] To H.T., Le Q.A., Bui H.T., Park J.-H., Kang D. (2023). Modulation of Spheroid Forming Capacity and TRAIL Sensitivity by KLF4 and Nanog in Gastric Cancer Cells. Curr. Issues Mol. Biol..

[145] Torisawa Y., Takagi A., Nashimoto Y., Yasukawa T., Shiku H., Matsue T. (2007). A Multicellular Spheroid Array to Realize Spheroid Formation, Culture, and Viability Assay on a Chip. Biomaterials.

[146] Uematsu N., Zhao Y., Kiyomi A., Yuan B., Onda K., Tanaka S., Sugiyama K., Sugiura M., Takagi N., Hayakawa A. (2018). Chemo-Sensitivity of Two-Dimensional Monolayer and Three-Dimensional Spheroid of Breast Cancer MCF-7 Cells to Daunorubicin, Docetaxel, and Arsenic Disulfide. Anticancer Res..

[147] Varan G., Akkın S., Demirtürk N., Benito J.M., Bilensoy E. (2021). Erlotinib Entrapped in Cholesterol-Depleting Cyclodextrin Nanoparticles Shows Improved Antitumoral Efficacy in 3D Spheroid Tumors of the Lung and the Liver. J. Drug Target..

[148] Vinci M., Gowan S., Boxall F., Patterson L., Zimmermann M., Court W., Lomas C., Mendiola M., Hardisson D., Eccles S.A. (2012). Advances in Establishment and Analysis of Three-Dimensional Tumor Spheroid-Based Functional Assays for Target Validation and Drug Evaluation. BMC Biol..

[149] Wan X., Li Z., Ye H., Cui Z. (2016). Three-Dimensional Perfused Tumour Spheroid Model for Anti-Cancer Drug Screening. Biotechnol. Lett..

[150] Wang Y., Wang J. (2014). Mixed Hydrogel Bead-Based Tumor Spheroid Formation and Anticancer Drug Testing. Analyst.

[151] Ware M.J., Keshishian V., Law J.J., Ho J.C., Favela C.A., Rees P., Smith B., Mohammad S., Hwang R.F., Rajapakshe K. (2016). Generation of an in Vitro 3D PDAC Stroma Rich Spheroid Model. Biomaterials.

[152] Wen Z., Liao Q., Hu Y., You L., Zhou L., Zhao Y. (2013). A Spheroid-Based 3-D Culture Model for Pancreatic Cancer Drug Testing, Using the Acid Phosphatase Assay. Braz. J. Med. Biol. Res..

[153] Wenzel C., Riefke B., Gründemann S., Krebs A., Christian S., Prinz F., Osterland M., Golfier S., Räse S., Ansari N. (2014). 3D High-Content Screening for the Identification of Compounds that Target Cells in Dormant Tumor Spheroid Regions. Exp. Cell Res..

[154] Weydert Z., Lal-Nag M., Mathews-Greiner L., Thiel C., Cordes H., Küpfer L., Guye P., Kelm J.M., Ferrer M. (2020). A 3D Heterotypic Multicellular Tumor Spheroid Assay Platform to Discriminate Drug Effects on Stroma versus Cancer Cells. SLAS Discov..

[155] Wu G., Zhan S., Rui C., Sho E., Shi X., Ding Y. (2019). Microporous Cellulosic Scaffold as a Spheroid Culture System Modulates Chemotherapeutic Responses and Stemness in Hepatocellular Carcinoma. J. Cell. Biochem..

[156] Wu K.W., Kuo C.-T., Tu T.-Y. (2021). A Highly Reproducible Micro U-Well Array Plate Facilitating High-Throughput Tumor Spheroid Culture and Drug Assessment. Glob. Chall..

[157] Xia H., Avci N.G., Akay Y., Esquenazi Y., Schmitt L.H., Tandon N., Zhu J.-J., Akay M. (2020). Temozolomide in Combination With NF-κB Inhibitor Significantly Disrupts the Glioblastoma Multiforme Spheroid Formation. IEEE Open J. Eng. Med. Biol..

[158] Xiong Q., Liu T., Ying Y., Yu X., Wang Z., Gao H., Lin T., Fan W., Zhang Z., Wei Q. (2024). Establishment of Bladder Cancer Spheroids and Cultured in Microfluidic Platform for Predicting Drug Response. Bioeng. Transl. Med..

[159] Yamawaki K., Mori Y., Sakai H., Kanda Y., Shiokawa D., Ueda H., Ishiguro T., Yoshihara K., Nagasaka K., Onda T. (2021). Integrative Analyses of Gene Expression and Chemosensitivity of Patient-Derived Ovarian Cancer Spheroids Link G6PD-Driven Redox Metabolism to Cisplatin Chemoresistance. Cancer Lett..

[160] Yoshida T., Sopko N.A., Kates M., Liu X., Joice G., Mcconkey D.J., Bivalacqua T.J. (2019). Impact of Spheroid Culture on Molecular and Functional Characteristics of Bladder Cancer Cell Lines. Oncol. Lett..

[161] Yu L., Ni C., Grist S.M., Bayly C., Cheung K.C. (2015). Alginate Core-Shell Beads for Simplified Three-Dimensional Tumor Spheroid Culture and Drug Screening. Biomed. Microdevices.

[162] Yu Q., Roberts M.G., Houdaihed L., Liu Y., Ho K., Walker G., Allen C., Reilly R.M., Manners I., Winnik M.A. (2021). Investigating the Influence of Block Copolymer Micelle Length on Cellular Uptake and Penetration in a Multicellular Tumor Spheroid Model. Nanoscale.

[163] Zhang J.Z., Bryce N.S., Lanzirotti A., Chen C.K.J., Paterson D., de Jonge M.D., Howard D.L., Hambley T.W. (2012). Getting to the Core of Platinum Drug Bio-Distributions: The Penetration of Anti-Cancer Platinum Complexes into Spheroid Tumour Models. Metallomics.

[164] Zhang J.Z., Bryce N.S., Siegele R., Carter E.A., Paterson D., de Jonge M.D., Howard D.L., Ryan C.G., Hambley T.W. (2012). The Use of Spectroscopic Imaging and Mapping Techniques in the Characterisation and Study of DLD-1 Cell Spheroid Tumour Models. Integr. Biol..

[165] Zhang X., Wang W., Yu W., Xie Y., Zhang X., Zhang Y., Ma X. (2005). Development of an in Vitro Multicellular Tumor Spheroid Model Using Microencapsulation and Its Application in Anticancer Drug Screening and Testing. Biotechnol. Prog..

[166] Zuchowska A., Kwapiszewska K., Chudy M., Dybko A., Brzozka Z. (2017). Studies of Anticancer Drug Cytotoxicity Based on Long-Term HepG2 Spheroid Culture in a Microfluidic System. Electrophoresis.

[167] Kim J.B., Stein R., O’Hare M.J. (2004). Three-Dimensional in Vitro Tissue Culture Models of Breast Cancer—A Review. Breast Cancer Res. Treat..

[168] Yau J.N.N., Adriani G. (2023). Three-Dimensional Heterotypic Colorectal Cancer Spheroid Models for Evaluation of Drug Response. Front. Oncol..

[169] Ahmad Zawawi S.S., Salleh E.A., Musa M. (2024). Spheroids and Organoids Derived from Colorectal Cancer as Tools for in Vitro Drug Screening. Explor. Target. Anti-Tumor Ther..

[170] Olabiran Y., Ledermann J., Marston N., Boxer G., Hicks R., Souhami R., Spiro S., Stahel R. (1994). The Selection of Antibodies for Targeted Therapy of Small-Cell Lung Cancer (SCLC) Using a Human Tumour Spheroid Model to Compare the Uptake of Cluster 1 and Cluster W4 Antibodies. Br. J. Cancer.

[171] Hulo P., Deshayes S., Fresquet J., Chéné A.-L., Blandin S., Boisgerault N., Fonteneau J.-F., Treps L., Denis M.G., Bennouna J. (2024). Use of Non-Small Cell Lung Cancer Multicellular Tumor Spheroids to Study the Impact of Chemotherapy. Respir. Res..

[172] Bassi G., Panseri S., Dozio S.M., Sandri M., Campodoni E., Dapporto M., Sprio S., Tampieri A., Montesi M. (2020). Scaffold-Based 3D Cellular Models Mimicking the Heterogeneity of Osteosarcoma Stem Cell Niche. Sci. Rep..

[173] Górnicki T., Lambrinow J., Golkar-Narenji A., Data K., Domagała D., Niebora J., Farzaneh M., Mozdziak P., Zabel M., Antosik P. (2024). Biomimetic Scaffolds—A Novel Approach to Three Dimensional Cell Culture Techniques for Potential Implementation in Tissue Engineering. Nanomaterials.

[174] Ware M.J., Colbert K., Keshishian V., Ho J., Corr S.J., Curley S.A., Godin B. (2016). Generation of Homogenous Three-Dimensional Pancreatic Cancer Cell Spheroids Using an Improved Hanging Drop Technique. Tissue Eng. Part C Methods.

[175] Białkowska K., Komorowski P., Bryszewska M., Miłowska K. (2020). Spheroids as a Type of Three-Dimensional Cell Cultures—Examples of Methods of Preparation and the Most Important Application. Int. J. Mol. Sci..

[176] Chaicharoenaudomrung N., Kunhorm P., Noisa P. (2019). Three-Dimensional Cell Culture Systems as an in Vitro Platform for Cancer and Stem Cell Modeling. World J. Stem Cells.

[177] Lewis N.S., Lewis E.E., Mullin M., Wheadon H., Dalby M.J., Berry C.C. (2017). Magnetically Levitated Mesenchymal Stem Cell Spheroids Cultured with a Collagen Gel Maintain Phenotype and Quiescence. J. Tissue Eng..

[178] Fattahi P., Rahimian A., Slama M.Q., Gwon K., Gonzalez-Suarez A.M., Wolf J., Baskaran H., Duffy C.D., Stybayeva G., Peterson Q.P. (2021). Core–Shell Hydrogel Microcapsules Enable Formation of Human Pluripotent Stem Cell Spheroids and Their Cultivation in a Stirred Bioreactor. Sci. Rep..

[179] Chan H.F., Zhang Y., Leong K.W. (2017). Efficient one-step production of microencapsulated hepatocyte spheroids with enhanced functions. Small.

[180] Gu Z., Fu J., Lin H., He Y. (2020). Development of 3D Bioprinting: From Printing Methods to Biomedical Applications. Asian J. Pharm. Sci..

[181] Dornhof J., Zieger V., Kieninger J., Frejek D., Zengerle R., Urban G.A., Kartmann S., Weltin A. (2022). Bioprinting-Based Automated Deposition of Single Cancer Cell Spheroids into Oxygen Sensor Microelectrode Wells. Lab Chip.

[182] Boularaoui S., Al Hussein G., Khan K.A., Christoforou N., Stefanini C. (2020). An Overview of Extrusion-Based Bioprinting with a Focus on Induced Shear Stress and Its Effect on Cell Viability. Bioprinting.

[183] Li J., Chen M., Fan X., Zhou H. (2016). Recent Advances in Bioprinting Techniques: Approaches, Applications and Future Prospects. J. Transl. Med..

[184] Kim M.H., Singh Y.P., Celik N., Yeo M., Rizk E., Hayes D.J., Ozbolat I.T. (2024). High-Throughput Bioprinting of Spheroids for Scalable Tissue Fabrication. Nat. Commun..

[185] Tian C., Tu Q., Liu W., Wang J. (2019). Recent Advances in Microfluidic Technologies for Organ-on-a-Chip. Cell Anal. Micronanofluid..

[186] Wongpakham T., Chungfong T., Jeamsaksiri W., Chessadangkul K., Bhanpattanakul S., Kallayanathum W., Tharasanit T., Pimpin A. (2024). Development of Pyramidal Microwells for Enhanced Cell Spheroid Formation in a Cell-on-Chip Microfluidic System for Cardiac Differentiation of Mouse Embryonic Stem Cells. Cells.

